# Immunological factors, but not clinical features, predict visceral leishmaniasis relapse in patients co-infected with HIV

**DOI:** 10.1016/j.xcrm.2021.100487

**Published:** 2021-12-30

**Authors:** Yegnasew Takele, Tadele Mulaw, Emebet Adem, Caroline Jayne Shaw, Susanne Ursula Franssen, Rebecca Womersley, Myrsini Kaforou, Graham Philip Taylor, Michael Levin, Ingrid Müller, James Anthony Cotton, Pascale Kropf

**Affiliations:** 1 Department of Infectious Disease, Imperial College London, London W2 1PG, UK; 2 Leishmaniasis Research and Treatment Centre, University of Gondar, PO Box 196, Gondar, Ethiopia; 3 Department of Metabolism, Digestion, and Reproduction, Imperial College London, London SW7 2AZ, UK; 4 Wellcome Sanger Institute, Wellcome Genome Campus, Hinxton CB10 1SA, UK

**Keywords:** Visceral leishmaniasis, HIV, CD4+, T cell counts, Interferon-gamma, PD1

## Abstract

Visceral leishmaniasis (VL) has emerged as a clinically important opportunistic infection in HIV patients, as VL/HIV co-infected patients suffer from frequent VL relapse. Here, we follow cohorts of VL patients with or without HIV in Ethiopia. By the end of the study, 78.1% of VL/HIV—but none of the VL patients—experience VL relapse. Despite a clinically defined cure, VL/HIV patients maintain higher parasite loads, lower BMI, hepatosplenomegaly, and pancytopenia. We identify three immunological markers associated with VL relapse in VL/HIV patients: (1) failure to restore antigen-specific production of IFN-γ, (2) persistently lower CD4^+^ T cell counts, and (3) higher expression of PD1 on CD4^+^ and CD8^+^ T cells. We show that these three markers, which can be measured in primary hospital settings in Ethiopia, combine well in predicting VL relapse. The use of our prediction model has the potential to improve disease management and patient care.

## Highlights

• Visceral leishmaniasis patients co-infected with HIV suffer from frequent VL relapse
• VL relapse is associated with low CD4^+^ T cell counts and low production of IFN-γ
• VL relapse is associated with increased PD1 expression on CD4^+^ and CD8^+^ T cells
• These three markers can predict whether and when these patients relapse

## Graphical abstract

**Figure:**
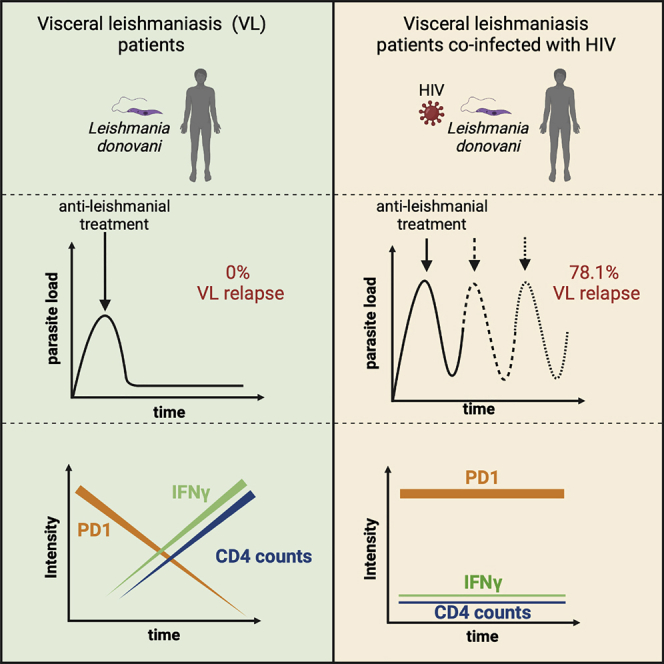

## Teaser

Patients with visceral leishmaniasis (VL) co-infected with HIV suffer from frequent VL relapse after successful anti-leishmanial treatment. Here, Takele et al. identify immune markers that can be used to predict when these patients will relapse. This has the potential to improve the disease management of these patients.

## Introduction

Visceral leishmaniasis (VL) is one of the most neglected tropical diseases. An estimated 550 million individuals are at risk of VL in high-burden countries, and 17,082 new cases of VL were reported in 2018.^1^ These numbers are widely acknowledged to underestimate the real burden because of the remote location of areas endemic for VL and the lack of surveillance. VL inflicts an immense toll on the developing world and impedes economic development, with an estimated annual loss of 2.3 million disability-adjusted life-years^2^ In Ethiopia, VL is one of the most significant vector-borne diseases; >3.2 million people are at risk of infection.^3^ VL is caused by infections with parasites of the *Leishmania donovani* species complex, but the majority of infected individuals control parasite replication and do not progress to disease. Some individuals will progress and develop VL, which is characterized by hepatosplenomegaly, fever, anemia, and wasting; this stage of the disease is generally fatal if left untreated.^4^^,^^5^ Following the HIV-1 pandemic, VL has emerged as an opportunistic infection: VL accelerates the progression of HIV infection to AIDS, and conversely, HIV infection increases the risk of developing symptomatic VL.^6^^,^^7^ Ethiopia has the highest rate of VL/HIV co-infections in Africa, with HIV present in up to 30% of VL cases.^8^

HIV co-infection present a major challenge in the prevention and control of VL^9^^,^^10^: VL/HIV co-infected patients experience higher rates of treatment failure, drug toxicity, mortality, and VL relapse rates compared to patients with VL alone.^10^^,^^11^ In Ethiopia, >50% of VL/HIV co-infected patients will experience relapse of VL between 3 and 9 months post-antileishmania treatment^12^. The mechanisms accounting for the increased rate of VL relapse in VL/HIV co-infected patients are poorly characterized. Markers such as low CD4^+^ T cell counts, high parasite loads at the time of VL diagnosis and during follow-up, not undergoing antiretroviral therapy (ART) at the time of VL diagnosis, and *Leishmania* antigenuria have shown variable degrees of prediction accuracy.^9^^,^^13^, ^14^, ^15^ Another predictive marker of VL relapse in VL/HIV co-infected patients is a history of VL relapse.^16^

One of the main immunological characteristics of VL patients is their profound immunosuppression.^17^ These patients do not respond to the Leishmanin skin test, their peripheral blood mononuclear cells (PBMCs) have an impaired capacity to produce IFN-γ and to proliferate in response to *Leishmania* antigen; this dysfunctional response to antigenic challenge is restored following successful chemotherapy^18^ and reviewed by Nylén and Sacks,^19^ Goto and Prianti,^20^ and Kumar and Nylén.^21^ The mechanisms leading to impaired T cell responses in VL patients remain to be fully understood.

Our knowledge of the immunopathology of VL/HIV co-infections is particularly sparse. Based on the current literature, it appears that the failure to control parasite replication results in chronic inflammation that leads to exhaustion of the immune system and failure to generate efficient T cell responses. Little is known about the immunological parameters associated with successful therapy. At the end of treatment, the discharge of VL/HIV co-infected patients from the hospital is based on clinical and parasitological cure.^22^ However, no clinical sign predicts an increased risk of relapse.^22^

Here, we followed VL and VL/HIV co-infected patients in Ethiopia and collected detailed clinical and immunological data during 12 months of follow-up. Genetic variation between parasites or re-infection of VL/HIV patients could be responsible for the increased rate of relapse. However, genomic data from isolates taken from the same patient cohorts as in the present study show that infections in VL and VL/HIV patients are caused by parasites from the same population and that almost all relapses are caused by recrudescence of the initial infection.^23^ Here, we aimed to generate the most detailed picture to date of the natural history of VL and VL/HIV infections in Ethiopia and to identify clinical and immunological markers associated with VL relapse. These markers need to be suitable for measurement in a primary hospital setting in Ethiopia, so that they could contribute to the improved evaluation of treatment success and ultimately improve poor outcomes for these patients.

## Results

### Clinical data

#### Frequency of VL relapse in VL and VL/HIV patients

**Figure 1:**
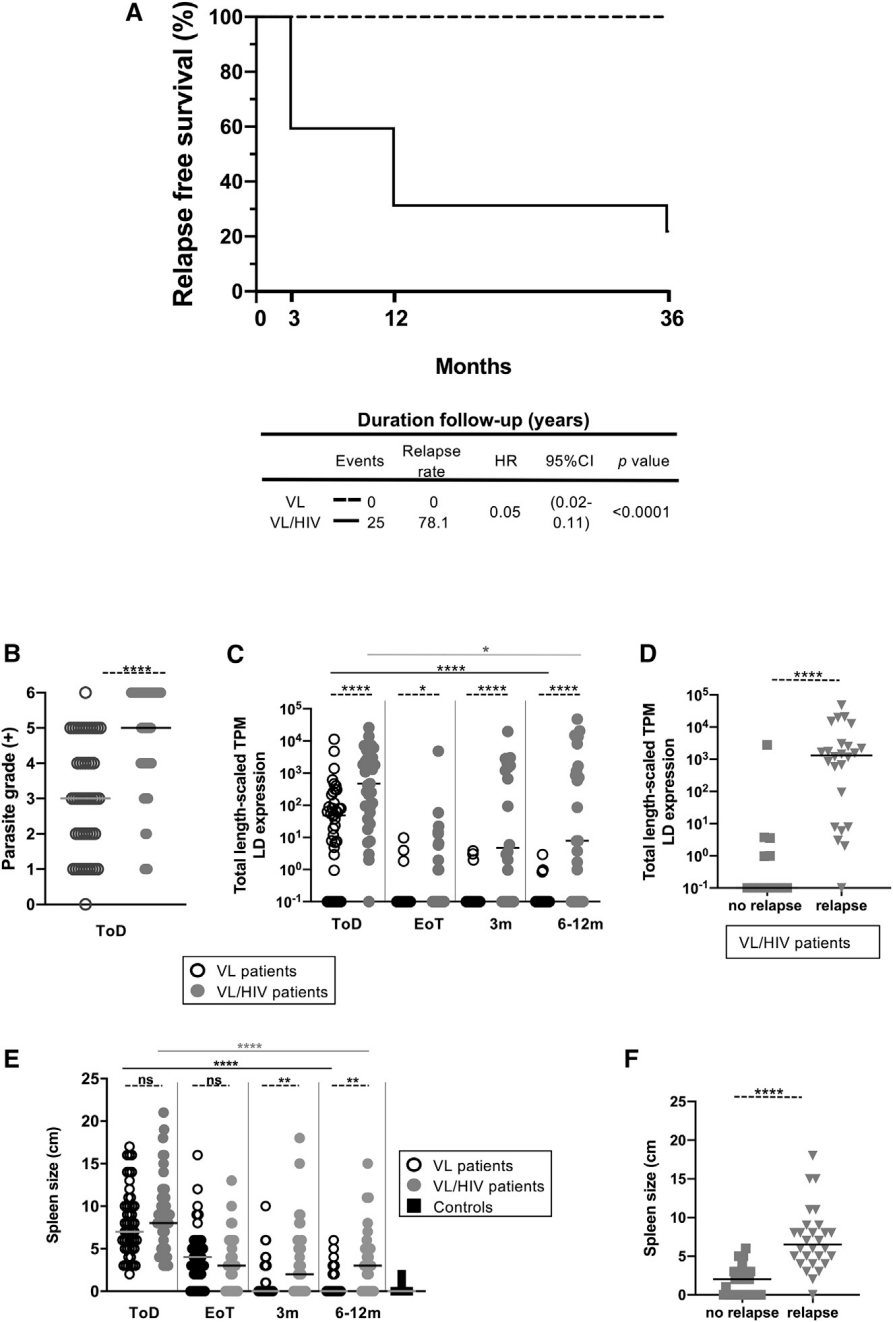
Relapse-free survival, parasite load, and spleen size (A) Kaplan-Meier curves of participant VL relapses comparing VL to VL/HIV patients. The hazard ratios (with 95% confidence intervals and p values) obtained from the Cox model indicated the change in survival following treatment for VL for these groups. HR, hazard ratio; CI, confidence interval. (B) Quantification of *Leishmania* amastigotes in smears of splenic aspirates collected from VL (n = 50) and VL/HIV (n = 49) patients at ToD. (C) Quantification of the total expression of *L. donovani* mRNA in blood from VL (ToD: n = 40, EoT: n = 31, 3 months: n = 31, 6–12 months: n = 37) and VL/HIV (ToD: n = 35, EoT: n = 30, 3 months: n = 24, 6–12 months: n = 25) patients during the 3- and 6- to 12-month follow-up periods. (D) Quantification of the total *L. donovani* mRNA expression in blood from VL/HIV patients who did not relapse (n = 13) and who relapsed (n = 24) after successful antileishmanial treatment. (E) Spleen size on VL (ToD: n = 50, EoT: n = 45, 3 months: n = 36, 6–12 months: n = 26), VL/HIV (ToD: n = 49, EoT: n = 39, 3 months: n = 32, 6–12 months: n = 26) patients and controls (n = 25). (F) Spleen size of VL/HIV patients who did not relapse (n = 28) and those who relapsed (n = 37) after successful antileishmanial treatment, during the 3- and 6- to 12-month follow-up periods. In (D) and (E), if a patient did not relapse during the 2 time points of follow-up and if a patient relapsed at both 3 and 6–12 months, this is represented as 2 measurements. Each symbol represents the value for 1 individual, the straight lines represent the median. Statistical differences between VL and VL/HIV patients at each time point or between no relapse and relapse were determined using a Mann-Whitney test; statistical differences between the 4 different time points for each cohort of patients were determined by the Kruskal-Wallis test. LD mRNA = *L. donovani* mRNA. ToD = time of diagnosis; EoT, end of treatment; ns, not significant; 3 m, 3 months post-EoT; 6–12 m, 6–12 months post-EoT;. See also Figure S1.

We followed VL/HIV patients for up to 3 years and compared their rates of VL relapse with those of VL patients. During this study, 3 VL/HIV patients left the treatment center before the end of treatment and 5 died during treatment; 41 were treated successfully (i.e., had a negative test of cure [TOC]). Following antileishmanial treatment, 9 were lost to follow-up and 32 VL/HIV patients were followed for up to 3 years; of these, 25 (78.1%) experienced at least 1 episode of VL relapse. This was in sharp contrast to VL patients; 4 VL patients died during treatment and 10 were lost to follow-up, but none of the successfully treated VL patients experienced VL relapse (Figure 1A).

The 46 VL patients who reached the end of the antileishmanial treatment responded well to their first course of treatment. In the VL/HIV group, 25 patients responded well to the first course of antileishmanial treatment, and had a negative TOC at the end of treatment, and were therefore discharged from the hospital. Sixteen VL/HIV patients responded poorly to the first line of treatment and had a positive TOC at end of treatment (EoT); they therefore required a longer course of antileishmanial drugs. We compared the duration of this initial treatment between VL/HIV patients who relapsed and those who did not relapse during follow-up: our results (Figure S1A) show that the duration of treatment was similar. Of the 16 patients who had a longer (>30 days) duration of treatment, 12 came back for follow-up and 4 were lost. As shown in Table S2, these 12 patients received different treatments.

#### Parasite grades and viral load

Parasite grades in VL/HIV patients at time of diagnosis (ToD) were significantly higher than those in VL patients (p < 0.001; Figure 1B), despite a similar duration of symptoms by patients in both groups (VL: 2.0 ± 0.2 versus VL/HIV: 2.0 ± 0.2 months, p > 0.05; Figure S1B). Since parasite grade is mainly measured at ToD, when the spleen is easily palpable, we used RNA sequencing (RNA-seq) to measure the total expression of *L. donovani* mRNAs (*Ld* mRNA) in blood (Figure 1C). In agreement with the parasite grades measured at ToD, there was significantly more *Ld* mRNA in VL/HIV patients; these levels decreased significantly in both cohorts of patients after antileishmanial treatment at EoT, but stayed significantly higher in VL/HIV patients. VL/HIV patients who relapsed during follow-up displayed significantly higher *Ld* mRNAs in blood than those without relapse (Figure 1D). Of note, by the end of our study, of the 5 VL/HIV patients who did not relapse but still had detectable *Ld* mRNA, 3 patients did not relapse and 2 were lost to follow-up (data not shown). There was no association in these 5 patients between the expression level of *Ld* mRNA and spleen sizes, interferon-γ (IFN-γ) level, and number of white and red blood cells and platelets (data not shown).

Measurement of plasma HIV-1 viral load showed that despite undergoing ART, 58.9% of VL/HIV patients still had detectable viral loads (Table 1, viral load [copies/mL]). There were no significant differences in either viral load (Table 1, viral load HIV mRNA) or total expression of HIV mRNA between time points (Table 1, viral load [copies/mL]) or between patients with and without relapse during follow-up (Table 1, HIV mRNA). There was no correlation between *Ld* mRNAs and viral loads in VL/HIV patients who relapsed during follow-up (p = 0.3356, data not shown); similar results were obtained with the correlation between the total expression of HIV-1 and *Ld* mRNAs (p = 0.0745, data not shown). Of note, there was no systematic pattern in how viral loads varied through follow-up (Figure S2A).

**Table 1:** Viral load, clinical symptoms, and liver and kidney function tests

	ToD	EoT	3 months	6–12 months	p
Viral load (copies/mL)					

VL/HIV	255 ± 402,369	150 ± 115,951	0.1 ± 61,395	0.1 ± 200,767	0.3632

Viral load H**I**V mRNA					

VL/HIV	673 ± 492,848	150 ± 62,366	75 ± 81,420	273 ± 141,025	0.4205

	No relapse	Relapse	p		

HIV mRNA					

VL/HIV	0.1 ± 154.1	397 ± 97,202	0.0575		

Fever					

VL	37.6 ± 0.2	36.0 ± 0.1	36.2 ± 0.1	36.0 ± 0.1	< 0.0001

VL/HIV	36.6 ± 0.1	36.4 ± 0.1	36.3 ± 0.2	36.3 ± 0.1	< 0.0001

p	0.0225	0.1502	0.4872	0.0233	

BMI					

VL	16.3 ± 0.2	16.7 ± 0.3	18.5 ± 0.2	19.1 ± 0.3	< 0.0001

VL/HIV	16.4 ± 0.3	17.0 ± 0.3	17.9 ± 0.3	17.1 ± 0.4	0.0010

p	0.7975	0.6094	0.0352	0.0050	

	Epistaxis (%)	Edema (%)	Concomitant infections (%)		

Other symptoms					

VL	9 (18.0)	13 (26.0)	15 (30.0)		

VL/HIV	11 (22.4)	4 (8.1)	14(28.6)		

	VL (%)	VL/HIV (%)			

Concomitant infections					

Pneumonia	7 (14.0)	6 (12.2)			

TB	2 (4.0)	5 (10.2)			

Intestinal parasites	2 (4.0)	0			

Malaria	1 (2.0)	2 (4.1)			

Herpes zoster	1 (2.0)	1 (2.0)			

Sepsis	1 (2.0)	0			

Viral hepatitis	1 (2.0)	0			

	ToD	EoT	p		

SGPT (<43 U/L)					

VL	34.0 ± 7.5	43.5 ± 5.7	ns		

VL/HIV	20.0 ± 2.1	28.5 ± 4.2	ns		

p	<0.001	0.002			

SGOT (<38 U/L)					

VL	62.0 ± 13.7	61.0 ± 5.2	ns		

VL/HIV	41.0 ± 4.9	35.0 ± 5.6	ns		

p	<0.001	<0.001			

BUN (4.7–23.5 mg/dL)					

VL	12.0 ± 1.9	9.0 ± 0.5	0.0022		

VL/HIV	12.4 ± 1.1	13.8 ± 1.1	ns		

p	ns	<0.001			

Creatinine (0.6–1.1 mg/dL)					

VL	0.9 ± 0.1	0.8 ± 0.1	0.0092		

VL/HIV	0.9 ± 0.2	1.0 ± 0.1	ns		

p	ns	<0.001			

HIV-1 viral load (copies/mL) in plasma from VL/HIV (ToD: n = 39, EoT: n = 33, 3 months: n = 27, 6–12 months: n = 21) patients. Viral load HIV mRNA: quantification of the total expression of HIV mRNA in blood from VL/HIV patients (ToD: n = 35, EoT: n = 30, 3 months: n = 24, 6–12 months: n = 25). HIV mRNA: quantification of the total HIV mRNA expression in blood from VL/HIV patients who did not relapse (n = 13) and who relapsed (n = 24) after successful antileishmanial treatment (3 and 6–12 months) during the 3- and 6- to 12-month follow-up periods. If a patient did not relapse during the 2 time points of follow-up and if a patient relapsed at both 3 and 6–12 months, this is represented as 2 measurements. Fever: body temperature was measured on VL (ToD: n = 49, EoT: n = 45, 3 months: n = 36, 6–12 months: n = 26) and VL/HIV (ToD: n = 49, EoT: n = 38, 3 months: n = 32, 6–12 months: n = 26) patients and controls (n = 25). Other symptoms: numbers and percentages of VL and VL/HIV patients presenting at ToD with epistaxis, edema, or concomitant infections. Concomitant infections: numbers and percentages of VL and VL/HIV patients presenting with the different concomitant infections. SGPT: serum glutamic oxaloacetic transaminase; SGPT, serum glutamic pyruvic transaminase; BUN, blood urea nitrogen; creatinine: creatinine was measured in the plasma of VL patients as described in Method details. The values in italic and in parentheses represent the normal values. Statistical differences between VL and VL/HIV patients at each time point were determined using a Mann-Whitney test, and statistical differences between the 4 different time points for each cohort of patients were determined by the Kruskal-Wallis test. ToD, time of diagnosis; EoT, end of treatment; ns, not significant; 3 m, 3 months post-EoT; 6–12 m, 6–12 months post-EoT.

#### Clinical presentation

The following clinical and laboratory data were collected from each patient before the start of antileishmanial therapy.

##### Fever

Results presented in Table 1 (fever) show that whereas both VL and VL/HIV patients had increased body temperatures at ToD (controls: 36.0°C ± 0.1°C, p < 0.0001), it was significantly lower in VL/HIV patients (p = 0.023) and decreased over time in both groups (p < 0.0001), but at 6–12 months was higher in VL/HIV patients as compared to VL patients (p = 0.023). There was no significant difference in the body temperature of VL/HIV patients with and without relapse during follow-up (data not shown).

##### Hepatosplenomegaly

As shown in Figure 1E, spleen sizes were similarly increased in both cohorts of patients at ToD and decreased at EoT. These continued to decrease in VL patients but stayed higher in VL/HIV patients at 3 and 6–12 months. Spleens were significantly more enlarged in VL/HIV patients who relapsed (Figure 1F). The liver was also palpable at ToD in 40.0% of VL and 63.3% of VL/HIV patients and was significantly more enlarged in VL/HIV as compared to VL patients (p = 0.012); the liver size decreased significantly at EoT (Figure S1C). There was no significant difference in liver size in VL/HIV patients with and without relapse during follow-up (data not shown).

##### Body mass index (BMI)

The median BMI of patients with VL and VL/HIV was below 18.5 at ToD (VL patients: 16.4 ± 0.3, VL/HIV patients: 16.7 ± 0.3; Table 1, BMI). The BMI of VL patients increased over time and at 3 months, was similar to those of controls (controls: 19.9 ± 0.6, p > 0.05); in contrast, the BMI of the VL/HIV patients stayed significantly lower compared to VL patients and controls over time (Table 1, BMI).

Of note, when compared at EoT, these clinical presentations were not significantly different in the VL/HIV groups that went on to relapse and those that did not (Table S3).

Epistaxis, edema, and concomitant infections are clinical symptoms that are routinely recorded at ToD in these patients. As shown in Table 1 (other symptoms), there were no differences between the number of patients experiencing epistaxis or co-infections (pneumonia, tuberculosis [TB], intestinal parasites, malaria, herpes zoster, sepsis, or viral hepatitis; Table 1, concomitant infections), but there were more VL patients presenting with edema.

Liver and kidney function are also routinely measured at ToD and EoT. Results show significantly lower levels of serum glutamic oxaloacetic transaminase (SGOT) and serum glutamic pyruvic transaminase (SGPT) in the VL/HIV groups as compared to the VL groups at both ToD and EoT (Table 1, SGOT and SGPT). The levels of blood urea nitrogen (BUN) and creatinine decreased significantly in the VL group at EoT but remained similar in the VL/HIV groups (Table 1, BUN and creatinine).

##### Hematological profile

White (WBC) and red blood cell (RBC) and platelet (PLT) counts were significantly decreased at ToD as compared to controls (Figures S3A, S3C, and S3E). At ToD, only PLT counts were higher in VL/HIV patients as compared to VL patients (Figure S3E). WBC counts increased in both groups at EoT, but thereafter only continued to increase in VL, but not in VL/HIV patients. Of note, WBC count levels in both VL and VL/HIV patients had not been restored 6–12 m post-EoT. Similarly, RBC and PLT counts increased in VL patients, and, in contrast to the counts in VL/HIV patients, were restored to levels similar to those of controls at EoT. During the follow-up of VL/HIV patients, WBC, RBC, and PLT counts were significantly lower in VL/HIV patients who relapsed as compared to those who did not relapse (Figures S3B, S3D, and S3F).

In summary, the results from the clinical data show that over time, VL/HIV patients maintained higher parasite loads, hepatosplenomegaly, and lower BMI, and remained pancytopenic as compared to VL patients.

### Immunological data

#### Antigen-specific production of IFN-γ and interleukin-10 (IL-10)

**Figure 2:**
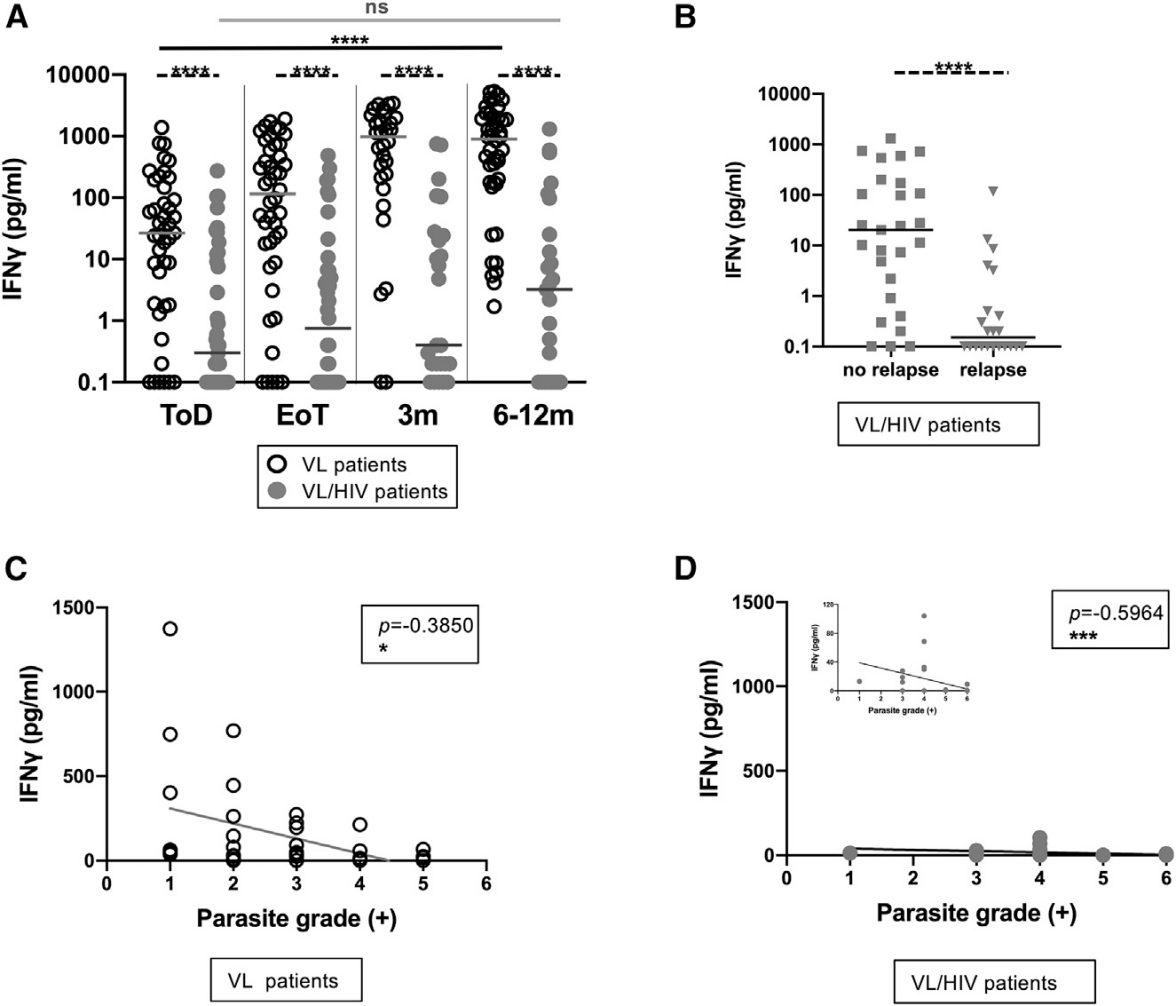
Whole blood assay: antigen-specific production of IFN-γ (A) Whole blood cells from VL (ToD: n = 43, EoT: n = 44, 3 months: n = 30, 6–12 months: n = 44) and VL/HIV patients (ToD: n = 39, EoT: n = 40, 3 months: n = 25, 6–12 months: n = 25) were cultured in the presence of soluble leishmania antigen (SLA), and IFN-γ was measured by ELISA in the supernatant after 24 h. (B) Comparison of the levels of antigen-specific IFN-γ produced by whole blood cells from VL/HIV patients who did not relapse (n = 27) and those who relapsed (n = 22) after successful antileishmanial treatment during the 3- and 6- to 12-month follow-up period. If a patient did not relapse during the 2 time points of follow-up and if a patient relapsed at both 3 and 6–12 months, this is represented as 2 measurements. (C and D) Correlation between parasite grades and IFN-γ at ToD in VL patients (n = 38) (C) and (D) VL/HIV patients (n = 34). Each symbol represents the value for 1 individual; the straight lines represent the median. Statistical differences between VL and VL/HIV patients at each time point or between no relapse and relapse were determined using a Mann-Whitney test; statistical differences between the 4 different time points for each cohort of patients were determined by the Kruskal-Wallis test and the correlation by the Spearman rank test.

Antigen-specific production of IFN-γ by whole blood cells was low at ToD in both cohorts of patients, but increased significantly in VL patients at EoT and was restored during follow-up (Figure 2A). In contrast, the levels of IFN-γ produced by whole blood (WB) cells from VL/HIV patients remained lower at all time points as compared to VL patients (Figure 2A). This was also true in the longitudinal follow-up of patients (Figures S2B and S2C, p = 0.0038 and p = 0.1682, respectively). We also compared the levels of antigen-specific IFN-γ in VL/HIV patients with and without relapse; in the longitudinal follow-up (Figure S2C), those who did not relapse produced significantly more IFN-γ (p = 0.0022). The median levels of IFN-γ produced by WB cells from patients who did not relapse after treatment were also significantly higher as compared to those who relapsed (Figure 2B) and were similar to those measured at ToD in VL patients (Figure 2A). There was a clear correlation between IFN-γ concentrations and parasite grade at ToD (Figures 2C and 2D).

IFN-γ production in response to phytohemagglutinin (PHA) followed the same pattern as antigen-specific stimulation, failing to recover from a lower level in VL/HIV patients as compared to VL patients (Table 2, VL: IFN-γ, VL/HIV: IFN-γ, and PHA: IFN-γ).

**Table 2:** Production of IFN-γ and IL-10 in response to SLA and PHA

	ToD	EoT	3 months	6–12 months	p
**VL: IFN-γ**					

SLA	28.4 ± 41.3	115.4 ± 80.4	972.2 ± 205.0	898.4 ± 229.6	<0.0001
PHA	30.6 ± 35.6	177.8 ± 123.0	190.8 ± 186.0	389.5 ± 196.7	0.0001
p	0.6728	0.6552	0.0637	0.1971	

**LV/HIV: IFN-γ**					

SLA	0.3 ± 7.8	0.8 ± 15.5	0.4 ± 40.5	3.2 ± 58.4	0.3411
PHA	6.4 ± 65.9	33.1 ± 129.1	14.5 ± 101.1	14.2 ± 148.6	0.5850
p	0.0001	0.0002	0.0249	0.0524	

**PHA: IFN-γ**					

VL	30.6 ± 35.6	177.8 ± 123.0	190.8 ± 186.0	389.5 ± 196.7	
VL/HIV	6.4 ± 65.9	33.1 ± 129.1	14.5 ± 101.1	14.2 ± 148.6	
p	0.1089	0.0764	0.0075	<0.0001	

**VL: IL-10**					

SLA	2.9 ± 1.3	0.1 ± 0.76	1.7 ± 1.3	0.3 ± 3.1	
PHA	26.9 ± 19.4	142.1 ± 30.2	648.9 ± 67.4	390.7 ± 38.7	
p	0.0002	<0.0001	<0.0001	<0.0001	

**VL/HIV: IL-10**					

SLA	0.3 ± 3.1	2.6 ± 1.8	0.7 ± 0.7	0.2 ± 1.2	0.1524
PHA	25.4 ± 16.9	227.3 ± 42.0	319.2 ± 41.6	255.5 ± 56.3	<0.0001
p	<0.0001	<0.0001	<0.0001	<0.0001	

**PHA: IL-10**					

VL	26.9 ± 19.4	142.1 ± 30.2	648.9 ± 67.4	390.7 ± 38.7	
VL/HIV	25.4 ± 16.9	227.3 ± 42.0	319.2 ± 41.6	255.5 ± 56.3	
p	0.1089	0.0764	0.0029	0.1076	

VL: IFN-γ: whole blood cells from VL patients (ToD: n = 43, EoT: n = 44, 3 months: n = 30, 6–12 months: n = 44) were cultured in the presence of SLA and PHA, and IFN-γ levels in the supernatants were measured by ELISA after 24 h.;VL/HIV: IFN-γ: whole blood cells from VL/HIV patients (ToD: n = 39, EoT: n = 40, 3 months: n = 25, 6–12 months: n = 25) were cultured in the presence of SLA and PHA, and IFN-γ levels in the supernatants were measured by ELISA after 24 h; PHA: IFN-γ: comparison of the levels of IFN-γ produced in response to PHA between VL and VL/HIV patients; VL: IL-10: whole blood cells from VL patients (ToD: n = 43, EoT: n = 44, 3 months: n = 30, 6–12 months: n = 44) were cultured in the presence of SLA and PHA, and IL-10 levels in the supernatant were measured by ELISA after 24 h; VL/HIV: IL-10: whole blood cells from VL/HIV patients (ToD: n = 39, EoT: n = 40, 3 months: n = 25, 6–12 months: n = 25) were cultured in the presence of SLA and PHA and IL-10 levels in the supernatant were measured by ELISA after 24 h. PHA: IL-10: comparison of the levels of IL-10 produced in response to PHA between VL and VL/HIV patients. Statistical differences between VL and VL/HIV patients or between SLA and PHA at each time point were determined using a Mann-Whitney test and statistical differences between the 4 different time points for each cohort of patients were determined by the Kruskal-Wallis test.

Antigen-specific production of IL-10 was lower in both groups as compared to PHA stimulation throughout follow-up (Table 2, VL: IFN-γ and VL/HIV: IFN-γ). The levels of IL-10 produced in response to PHA were similar in VL and VL/HIV at ToD, EoT, and 6–12 months (Table 2, PHA: IFN-γ). Sustained levels of antigen-specific IL-10 production by WBCs are thus not associated with disease severity.

#### CD4^+^ and CD8^+^ T cell counts in VL and VL/HIV co-infected patients

**Figure 3:**
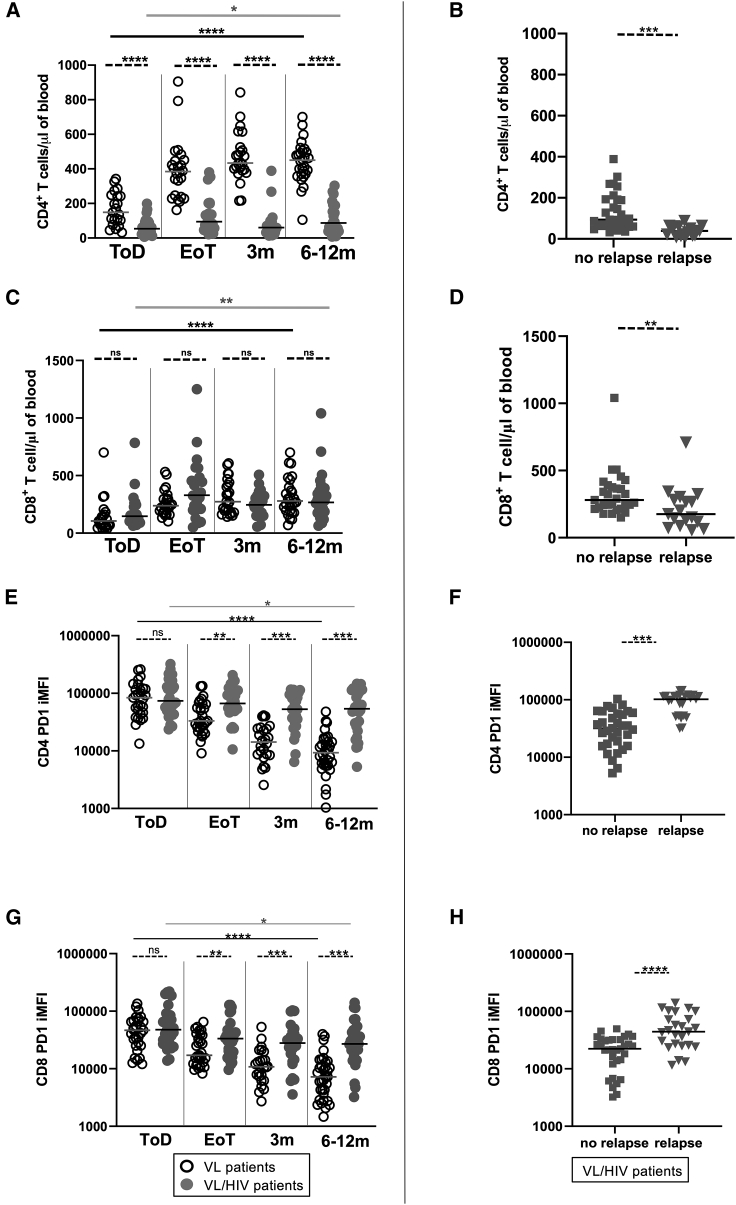
CD4^+^ and CD8^+^ T cell counts and PD1 expression (A and C) CD4^+^ T cell counts (A) and (C) CD8^+^ T cell counts were measured by flow cytometry in the blood of VL (ToD: n = 21, EoT: n = 24, 3 months: n = 24, 6–12 months: n = 28) and VL/HIV patients (ToD: n = 27, EoT: n = 24, 3 months: n = 21, 6–12 months: n = 25). (B and D) Comparison of CD4^+^ T cell counts (B) and (D) CD8^+^ T cell counts in VL/HIV patients who did not relapse (n = 29) and those who relapsed (n = 17) during the 3- and 6- to 12-month follow-up periods. (E and G) CD4 PD1 iMFI (E) and (G) CD8 PD1 iMFI was measured by multiplying the percentage of CD4^+^ T cells or CD8^+^ T cells and the median fluorescence intensity of PD1 as measured by flow cytometry in the PBMCs of VL (ToD: n = 29, EoT: n = 29, 3 months: n = 24, 6–12 months: n = 34) and VL/HIV patients (ToD: n = 28, EoT: n = 32, 3 months: n = 26, 6–12 months: n = 32). (F and H) Comparison of CD4 PD1 iMFI (F) and (H) comparison of CD8 PD1 iMFI in the blood of VL/HIV patients who did not relapse (n = 33) and those who relapsed (n = 25) during the 3- and 6- to 12-month follow-up periods. (B, D, F, and H) If a patient did not relapse during the 2 time points of follow-up and if a patient relapsed at both 3 and 6–12 months, this is represented as 2 measurements. Each symbol represents the value for 1 individual; the straight lines represent the median. Statistical differences between VL and VL/HIV patients at each time point or between no relapse and relapse were determined using a Mann-Whitney test, and statistical differences between the 4 different time points for each cohort of patients were determined by the Kruskal-Wallis test.

To determine whether the lower production of IFN-γ at ToD in VL patients as compared to 6–12 months and in VL/HIV as compared to VL patients are associated with low T cell counts, the absolute CD4^+^ T cell counts were measured in both cohorts of patients (Figure 3A). CD4^+^ T cell counts in VL patients were lower at ToD but were restored at EoT (controls CD4^+^ T cell counts: 455 ± 16.4 cells/μL of blood; data not shown). Despite an increase in CD4^+^ T cell counts at EoT in VL/HIV patients, CD4^+^ T cell counts remained significantly lower at all time points as compared to VL patients (Figure 3A). Longitudinal follow-up of individual patients also shows a significant increase over time in CD4^+^ T cell counts in VL, but not in VL/HIV patients (Figures S2D and S2E, p = 0.0069 and p = 0.0923, respectively). Median CD4^+^ T cell counts in patients who did not relapse after treatment was significantly higher as compared to those who relapsed (Figure 3B).

Results presented in Figure 3C show that CD8^+^ T cell counts increased over time (p < 0.05), but that there were no significant differences between VL and VL/HIV patients at any time point (p > 0.05). CD8^+^ T cell counts were significantly lower (VL: p < 0.0001; VL/HIV: p = 0.004) at ToD as compared to controls (controls CD8^+^ T cell counts: 293.0 ± 25.4 cells/μL of blood; data not shown), but were restored to control levels in both groups at EoT (p < 0.05). The median CD8^+^ T cell counts in patients who did not relapse after treatment was significantly higher as compared to those who relapsed (Figure 3D).

#### Inflammatory cytokines in VL and VL/HIV patients

It is well established that markers of inflammation are high in the plasma of VL patients,^19^ and the levels of C-reactive protein (CRP) were elevated at ToD and fell to physiological levels at the end of the follow-up (Figure S4A).^24^ In contrast to VL, in VL/HIV patients, these levels decreased over time, but remained significantly higher (Figure S4A).

The levels of tumor necrosis factor-α (TNF-α), IL-8, and IL-6 were higher in the plasma of both cohorts of patients at ToD as compared to the other time points (Figures S4B–S4D). These levels decreased in both groups at EoT, and continued to decrease over time in the VL, but not in the VL/HIV cohort. The levels of IFN-γ were significantly higher at ToD in the plasma of VL patients and dropped to levels that were similar to those of VL/HIV patients during the follow-up (Figure S4E). IL-1β was only detectable at ToD in the VL group and dropped to levels below detection limit at EoT; IL-12 levels were below detection limits in the large majority of the plasma (data not shown). IL-10 levels were higher at ToD in the plasma of VL patients and decreased at EoT. At both time points, they remained significantly higher as compared to the VL/HIV group (Figure S4F), and continued to decrease in the VL group after 3 and 6–12 months, but remained significantly higher in the VL/HIV group. The levels of the anti-inflammatory cytokines IL-4 and IL-13 were largely below detection limits (data not shown).

Since type I IFNs can promote parasite growth ^25^, we assessed whether the levels of IFN-α and IFN-β were higher in the plasma of VL/HIV patients who relapsed as compared to those who did not relapse during follow-up. Our results show no significant difference between the two groups (Table S4).

#### Inhibitory receptors expressed by CD4^+^ and CD8^+^ T cells from VL and VL/HIV co-infected patients

Immune checkpoint molecules can trigger immunosuppressive signaling pathways that can drive T cells into a state of hyporesponsiveness, with reduced effector functions and sustained expression levels of inhibitory receptors.^26^ Here, we focused on CTLA-4 and PD1 expression on CD4 and CD8^+^ T cells. CTLA-4 expression on T cells from VL, VL/HIV patients, and controls was below the detection limit (<1%, data not shown). PD1 integrated median fluorescence intensity (iMFI) of CD4^+^ T cells was higher at ToD as compared to the other time points in both cohorts of patients (Figure 3E), but decreased significantly in VL patients at EoT and was restored at 3 months (controls: CD4 PD1 iMFI: 12,737 ± 3,095, p > 0.05, data not shown). In contrast, CD4 PD1 iMFI in VL/HIV patients remained higher after treatment and during follow-up (Figure 3E). The cross-sectional pattern was replicated in longitudinal trends for individual patients (Figures S2F and S2G, p = 0.0002 and p = 0.0057, respectively). The expression levels of CD4 PD1 iMFI were significantly lower in VL/HIV patients who did not relapse after treatment as compared to those who did relapse (Figure 3F).

CD8 PD1 iMFI was higher at ToD as compared to the other time points in both cohorts of patients (Figure 3G) but were restored at EoT in VL patients (controls: CD8 PD1 iMFI: 14144 ± 1829, p > 0.05; data not shown). In contrast, CD8 PD1 iMFI remained higher in VL/HIV patients as compared to controls and VL patients after treatment and during follow-up (Figure 3G). The expression levels of CD8 PD1 iMFI were significantly lower in VL/HIV patients who did not relapse after treatment as compared to those who relapsed (Figure 3H).

In summary, our study has identified 3 immunological markers that are associated with VL relapse in VL/HIV patients and that are expressed at different levels as compared to VL patients: lower CD4^+^ T cell count, lower IFN-γ production in the whole blood assay (WBA), and higher PD1 expression on CD4^+^ T cells.

#### Prediction of relapse

Next, we formally tested whether the clinical and immunological parameters we measured at the end of treatment could be used to predict if and when the patients will relapse. Since none of the clinical parameters were significantly different at EoT between VL and VL/HIV patients, we tested whether the 3 immunological parameters we identified could be used as predictors of relapse. The results of the multinomial logistic regression analysis are summarized in Table 3.

**Table 3:** Multinomial regression to predict time of relapse in VL/HIV patients

	Dependent variable	
	Relapse at 3 months (1)	Relapse at 6–12 months (2)
CD4	0.071∗∗∗ (0.000)	−0.164∗∗∗ (0.000)
IFN-γ	0.044∗∗∗ (0.000)	−1.321∗∗∗ (0.000)
PD1	0.0001∗∗∗ (0.000)	0.00003∗∗∗ (0.00000)
CD4:IFN-γ	0.002∗∗∗ (0.000)	0.010∗∗∗ (0.000)
CD4:PD1	0.00000∗∗∗ (0.00000)	0.00000∗ (0.00000)
IFN-γ:PD1	0.00000∗∗∗ (0.00000)	0.00000∗∗∗ (0.00000)
CD4:IFN-γ:PD1	0.00000∗∗∗ (0.000)	0.00000∗∗∗ (0.00000)
Constant	−9.553∗∗∗ (0.000)	8.897∗∗∗ (0.000)
Akaike Inf. Crit.	53.005	53.005

Results of a multinomial logistic regression to predict the relapse of VL/HIV individuals. CD4 (CD4^+^ T cell counts), IFN-γ (WBA), and PD1 (CD4 PD1 iMFI) levels from EoT were used as predictors in predicting relapse at 3 potential time points; relapse at 3 months, relapse at 6–12 months, or no relapse recorded within 12 months of EoT. ∗p < 0.1; ∗∗p < 0.05; ∗∗∗p < 0.01.

**Figure 4:**
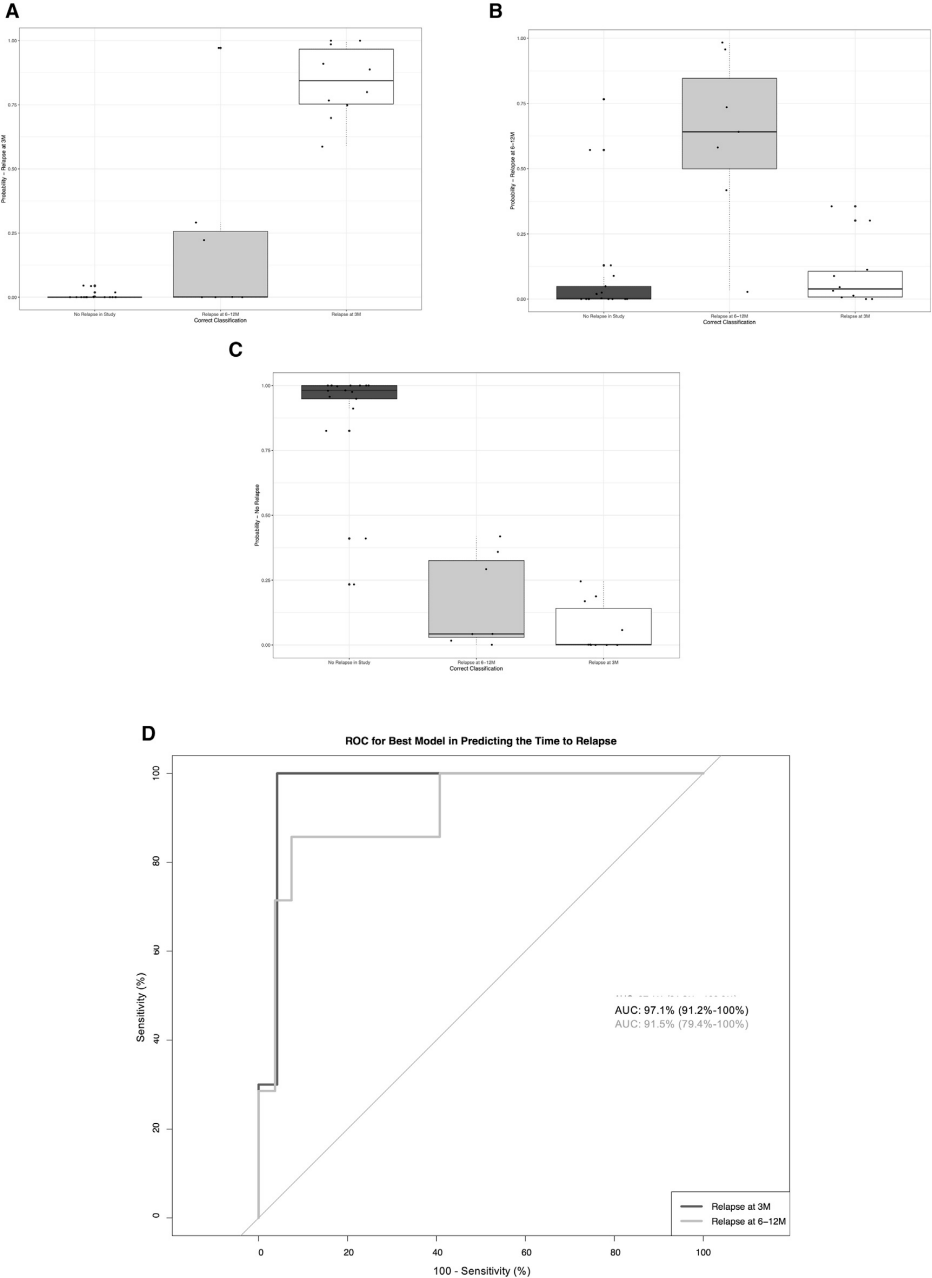
Performance of multinomial logistic regression model in predicting relapse time for VL/HIV individuals (A) Performance of model in predicting relapse at 3 months from EoT. (B) Performance of model in predicting relapse at 6–12 months from EoT. (C) Performance of model in predicting VL/HIV individuals who do not relapse with VL within 12 months of study from EoT. (D) Receiver operating characteristic (ROC) for predicting VL relapse at 3 months and relapse at 6–12 months groups for VL/HIV individuals from logistic regression model. See also Figure S5.

We trained a multinomial logistic regression model to predict early (at the 3-month follow-up), later (at 6–12 months), or no relapse 12-month follow-up, based on CD4 T cell counts, IFN-γ and PD1 levels at EoT. This model performs well at distinguishing relapse times (Figure 4; relapse at 3 months area under the curve [AUC] = 0.971, 95% confidence interval [CI] = 0.912–1.00; relapse at 6–12 months; AUC = 0.915, CI = 0.794–1.00) and particularly in distinguishing early relapse compared to relapse at later time points, which will be critical in identifying patients who need continuing treatment.

Since spleen size has been associated with VL relapse,^27^ we also assessed how well variation in spleen size predicted time to relapse (Figures S5A and S5B); this achieved an AUC of 0.612, CI = 0.38–0.844, and AUC 0.662 (CI = 0.428–0.896) for predicting relapse at 3 months and 6–12 months, respectively. We performed a DeLong’s test between spleen size and our best model and found that our best model performed significantly better than spleen size in predicting relapse within 3 months of EoT (p < 0.005).

Having found a significant difference in *Ld* mRNA between relapse status of VL/HIV at EoT, we also tested the ability of *Ld* mRNA in predicting time to relapse (Figures S5C and S5D). This produced an AUC of 0.769 (CI = 0.578–0.959) and 0.586 (0.361–0.811) for relapse at 3 and 6–12 months, respectively. We also performed a DeLong’s test between *Ld* mRNA and our best model, and similarly to spleen size found that our best model performed significantly better than *Ld* mRNA in predicting relapse within 3 months of EoT (p = 0.04). Despite a relatively good performance of *Ld* mRNA in predicting relapse within 3 months here, we did not include this parameter in our best model due to the impracticality of measuring this marker in the given hospital setting to maximize the clinical translation of our relapse model.

When assessing the predictive potential of the variables measured in this study, we tested for significant differences between each immunological/clinical marker at EoT between relapse statuses for VL/HIV individuals (Table S3). Aside from *Ld* mRNA (p = 0.0022), we found no other variables to be significantly different between relapse status for VL/HIV. In addition, none of these parameters had significant predictive value within logistic regression modeling in predicting relapse outcome (p > 0.05, data not shown) alone, and our current sample size restricted the use of multiple parameters in multinomial modeling in this instance.

## Discussion

The rate of VL/HIV co-infection is very high in Ethiopia and represents a major public health problem mainly due to the high rate of VL relapse in these patients. Currently, there is no means to assess the probability of VL relapse after successful treatment. In this study, we followed cohorts of VL and VL/HIV patients for up to 3 years, including detailed immunological follow-up for up to 12 months. This detailed follow-up reveals a higher rate of relapse than previously identified,^12^ with 78.1% of VL/HIV patients experiencing at least 1 relapse during the follow-up. Since 9 VL/HIV patients were lost to follow-up in our study, it is possible that the relapse rate could be even higher. This higher relapse rate is likely to be due to the longer duration of our study and emphasizes the need for longer clinical follow-up of these patients.

Our results show that VL/HIV patients harbor a higher parasite load in splenic aspirates than VL patients at ToD. Quantifying parasite load in splenic aspirates over time in VL and VL/HIV patients has been an ongoing challenge, as it is not possible to perform splenic aspirates when the spleen size is <3 cm below the costal margin. To evaluate the parasite loads in these patients over time, we measured the total expression of *Ld* mRNA in whole blood. This is the first study that compares parasite loads in VL and VL/HIV patients before and after treatment and over time. Parasite gene expression in blood was significantly correlated with spleen parasite load at ToD (p < 0.0001, r = 0.7597; data not shown), suggesting that measuring the total mRNA in whole blood is a good alternative to splenic aspirate. A recent study showed a strong correlation between tissue parasite burden and blood parasite load measured by qPCR.^28^ While regular monitoring of parasite load is likely to be a valuable means to predict relapse, especially in VL/HIV patients, neither qPCR nor RNA-seq are available in many of the leishmaniasis treatment centers in Ethiopia; therefore, alternative tests that can be performed in these settings are needed.

Despite being on ART, 58% of co-infected patients had a detectable HIV viral load; 47 were on first-line treatment and 2 were on second-line treatment. The high incidence of detectable viral load in this population could be due to resistance to HIV drugs, which has been reported to both first- and second-line treatments^29^; poor availability of ART for the population of migrant workers during the agricultural season; or poor adherence to ART. While viral load in this population of HIV patients should be monitored more closely, VL/HIV patients who relapsed after successful antileishmanial treatment had similar viral load in plasma as those who did not relapse, and there was no correlation between the viral load and total *Ld* mRNA expression. In agreement with previous studies, this suggests that VL relapse in VL/HIV patients occurs independently of the viral load,^13^^,^^30^, ^31^, ^32^ but we cannot exclude that better long-term management of HIV in these patients could reduce the VL relapse rate.

As previously shown,^33^ both VL and VL/HIV patients have a lower BMI at ToD as compared to the next time points. The follow-up of VL patients revealed that their BMI is only restored at the last time point, 6–12 months after the end of the antileishmanial treatment. In contrast, the BMI of VL/HIV patients remained below 18.5 throughout follow-up. It is generally accepted that malnutrition plays a key role in increased susceptibility to infection and/or disease severity by weakening both innate and acquired immunity.^34^^,^^35^ It is therefore possible that a low BMI contributes to poor prognosis of VL/HIV patients. The higher levels of markers of inflammation (Figure S4) as compared to those in VL patients in their plasma may also contribute to their inability to regain weight as these cytokines have been shown to suppress appetite.^36^ Better management of malnutrition in these patients may also help to improve their ability to mount appropriate immune responses.

Severe pancytopenia is a hallmark of visceral leishmaniasis. It is thought to reflect bone marrow suppression and splenic sequestration. VL patients suffer from ineffective hematopoiesis; reticulo-endothelial cells infiltrate the bone marrow that becomes hyperplasic,^37^^,^^38^ and *L. donovani* can also establish infection in the bone marrow. In addition to bone marrow failure, spleen sequestration is thought to play a major role in the severe pancytopenia observed in these patients.^38^^,^^39^ HIV infection is also associated with pancytopenia and bone marrow failure.^40^^,^^41^ Whereas in VL patients the number of WBC and RBC and platelets is at least partially restored over time, this was not the case in the VL/HIV co-infected cohort. Both pathogens can infect hematopoietic stem/progenitor cells (HSPCs), and, at least in HIV infection, this can interfere with hematopoiesis.^42^ It is possible that the combined impact of *L. donovani* and HIV, via direct infection of HSPCs, or indirectly, via mechanisms such as high levels of chronic inflammation, becomes too detrimental for an adequate bone marrow output.

In summary, our clinical data suggest that better management of malnutrition and ART could improve the immune responses of VL/HIV patients and result in a more efficient control of parasite replication. In addition to the follow-up of the clinical natural history of VL and VL/HIV infections, we also measured immunological markers to identify (1) parameters resulting in VL relapse and (2) predictors of relapse. Since VL occurs in remote areas in Ethiopia, we focused on markers that can be tested in a primary hospital setting.

Our results using a WBA to measure the production of antigen-specific IFN-γ show that in contrast to those of VL patients, WBC from VL/HIV patients remain hyporesponsive, as they fail to restore their capacity to produce IFN-γ over time. The levels of IFN-γ produced by WBC from VL/HIV patients without relapse after treatment was significantly higher as compared to those with relapse; however, these values were similar to those of VL patients at ToD, a time when these patients cannot control parasite replication. In the WBA, CD4^+^ T cells are the main source of IFN-γ, and this IFN-γ has been shown to contribute to parasite killing.^43^^,^^44^I It is therefore tempting to speculate that the inability of CD4^+^ T cells in VL/HIV patients to produce antigen-specific IFN-γ over time plays a major role in the absence of efficient control of parasite replication after treatment. We propose that the following mechanisms may play a role in the suboptimal production of IFN-γ:

(1) Low production of antigen-specific IFN-γ is caused by low numbers of CD4^+^ T cells: our results show that VL/HIV patients fail to achieve normal CD4^+^ T cell counts. Since CD4^+^ T cells have been shown to be the main IFN-γ-producing cells in the WBA,^43^^,^^44^ we propose that the impaired antigen-specific production of IFN-γ in VL/HIV patients is at least in part due to lower CD4^+^ T cell counts as compared to VL patients. In contrast, CD4^+^ T cell counts were restored in VL patients after treatment. This shows that there is a normal recovery of CD4^+^ T cell counts in patients who are only infected with *Leishmania* parasites. It has been shown that despite ART, poor recovery of CD4^+^ T cell counts is common, especially in individuals who start ART with low CD4^+^ T cell counts^45^, ^46^, ^47^; and results of a multi-country prospective cohort study in sub-Saharan Africa showed a suboptimal recovery of CD4^+^ T cells despite sustained viral suppression on continuous first-line ART.^48^ In addition to bone marrow failure, reduced thymic output may play a role in the lower number of CD4^+^ T cells, and indeed, a recent study showed a clear correlation between decreased numbers of recent thymic emigrants and poor CD4^+^ T cell recovery in HIV patients.^49^ The study by Silva-Freitas et al.^50^ also suggested that in VL/HIV co-infected patients who do not relapse, more new emigrant T cells can be detected that may contribute to the control of parasite replication. In addition to its potential contribution to the lower levels of IFN-γ produced in the WBA, a poor CD4^+^ T cell recovery is associated with persistent immune activation and inflammation^51^ that in turn contribute to higher risks of several morbidities as well as death.^52^^,^^53^ Whereas to date, there are no effective alternative ART treatments that have been shown to increase the restoration of CD4^+^ T cell counts, it has been shown that early initiation of ART may boost the restoration of CD4^+^ T cell counts; furthermore inclusion of dolutegravir in first-line treatment could improve further CD4^+^ T cell recovery^54^ through more rapid and sustained suppression of HIV replication. It also has a higher barrier to resistance, making treatment failure less likely. In VL patients, high parasite burden is also associated with lower CD4^+^ T cell counts. This is significantly improved after successful treatment.^55^ It is likely that similar to what is observed in HIV patients, high levels of inflammation, bone marrow failure, reduced thymic output, and splenic sequestration contribute to the lower CD4^+^ T cells counts in VL patients. It is therefore difficult to distinguish the impact of HIV and/or *L. donovani* on the lower CD4^+^ T cells counts in VL/HIV patients.
Low CD4^+^ T cell counts could also be due to increased frequencies of apoptotic CD4^+^ T cells. It has been shown that in a mouse model of infection with *L. donovani*, IFN-γ^+^ CD4^+^ T cells died by apoptosis via TLR7-mediated IRF-5 upregulation of DR5; the authors suggested that this was triggered by tissue disruption.^56^ Whereas damage to the spleen architecture in VL has been described mainly in animal and experimental models, at least one study showed profound damage to the spleen.^57^ It is therefore possible that this intense tissue destruction results in CD4^+^ T cell apoptosis.
  (2) Persistent inflammation contributes to T cell dysfunction and the subsequent lower production of IFN-γ.^26^ It has been well established that persistent inflammation remains in HIV patients despite viral suppression and an increase in CD4^+^ T cell counts.^58^, ^59^, ^60^ *Leishmania* infections alone are also associated with persistent inflammation, and the present study shows that levels of cytokines such as TNF-α, IL-6, and IL-8 remained increased 6–12 months after the end of antileishmanial treatment. In our cohort of VL/HIV patients, the levels were even further increased. It is therefore likely that this persistent inflammation contributes to T cell dysfunction.
    (3) Lower production of IFN-γ is due to T cell exhaustion. In addition to inflammation, persistent antigenic stimulation results in T cell exhaustion, characterized by a progressive loss of efficient effector functions and co-expression of inhibitory receptors.^26^^,^^61^^,^^62^ Here, the immune checkpoint molecule PD1 was clearly expressed on CD4^+^ and CD8^+^ T cells from both patient cohorts at diagnosis. Since PD1 is induced in T cells following activation by different signals such as IL-2, IL-7, type I IFNs, and signaling via the T cell receptor (TCR), it is specifically a marker of T cell activation.^63^ During chronic infection, the levels of PD1 are sustained and are associated with T cell dysfunction. However, whereas high expression of PD1 is mainly associated with T cell downregulation,^62^ we cannot exclude that PD1 expression is a sign of T cell activation due to the higher levels of parasites persisting in these patients as compared to VL patients. PD1 levels fell to be similar to those of controls in VL patients, accompanied by increasing levels of IFN-γ secretion by whole blood cells, but remained higher throughout follow-up in VL/HIV co-infected patients as compared to VL patients, with lower levels of antigen-specific IFN-γ produced by whole blood cells. CD4^+^ and CD8^+^ T cells in VL/HIV patients thus continue to have hallmarks of exhausted cells; they express PD1 and produce lower levels of IFN-γ as compared to those from VL patients. Exhausted T cells do not lose all of their effector functions—they become hypofunctional and can still proliferate and produce effector molecules.^26^^,^^61^^,^^62^ Whereas exhaustion is seen mainly as a dysfunctional state, exhausted T cells have been shown to at least partially contain chronic infections and play an important role in limiting immunopathology.^26^^,^^61^^,^^62^ It is therefore possible that during VL infections, activated T cells progressively become exhausted and that while still contributing to the control of parasite replication, they also contribute to prevent tissue damage. After successful treatment, with the clearance of the large majority of parasites and a progressively reduced inflammation, our results show that the frequency of these exhausted cells is restored to control levels. In contrast, in VL/HIV patients, the persistent high antigenic stimulation and inflammation contribute to the maintenance of exhausted T cells.
      (4) Whereas we have shown that sustained antigen-specific IL-10 production in the WBA was not associated with disease severity in patients from northwest Ethiopia, we and others have shown that high levels of IL-10 are present in the plasma of VL patients.^19^ IL-10 has been shown to promote parasite growth in splenic aspirate cell cultures, as neutralization of IL-10 resulted in higher production of IFN-γ and increased parasite killing.^64^ Therefore, we cannot exclude that the higher levels of IL-10 in the plasma of VL and VL/HIV patients, as compared to controls, also contribute to the impaired production of IFN-γ in the WBA. However, in the WBA that we performed with the blood from VL patients, IL-10 production in response to PHA increased significantly over time. This was mirrored by IFN-γ production, suggesting that even in the presence of high levels of IL-10, high levels of IFN-γ can still be produced.

We propose that in VL patients at ToD, lower CD4^+^ T cell counts and T cell exhaustion measured at ToD as compared to the next time points play key roles in the impaired production of IFN-γ, but that this is reversed at the end of successful antileishmanial treatment. In contrast, in VL/HIV patients, CD4^+^ T cell counts remain lower as compared to VL patients and exhausted after antileishmanial treatment, resulting in severely impaired production of IFN-γ that contributes to uncontrolled parasite replication and frequent relapses seen in VL/HIV patients.

Our study has identified 3 markers that were measured in a primary care hospital in Ethiopia that are associated with VL relapse in VL/HIV patients and that were differentially expressed at EoT as compared to VL patients: lower CD4^+^ T cell counts, lower IFN-γ production in a WBA, and higher PD1 expression levels on CD4^+^ T cells. The multinomial logistic regression analyses show good differentiation between all 3 groups (relapse at 3 months, relapse at 6–12 months, and no relapse). Perhaps most clinically translatable is the clear distinction seen between the 3- and 6- to 12-month groups. Importantly, these 3 measurements combine non-additively in the best prediction model (relapse at 3 months), suggesting that these factors interact in driving relapse in VL/HIV patients. It must be noted that even though all of these markers were measured in a primary care hospital setting in Ethiopia, the material required to perform these experiments may not always be readily available in other primary care hospitals. However, these results provide a promising indication of the potential of widely available predictors from blood immunology tests to identify VL/HIV co-infected individuals who are most at risk of early relapse and need to be further validated in external cohorts of patients to ensure reproducibility. The simplicity of the prediction model will also lend itself readily to translation into a formula that can be calculated on site. This is extremely promising for such a setting, where individuals who are most at risk of relapse within 3 months could be identified and treated further. For these patients, in addition to an optimization of their ART treatment and an improvement in their nutritional status, a longer antileishmanial treatment may be beneficial. Furthermore, the impaired production of IFN-γ and higher expression of PD1 throughout follow-up in VL/HIV as compared to VL patients suggest that immunotherapy through IFN-γ administration^65^ and/or PD1/PDL-1 blockade may improve parasite killing and disease control in these patients.

### Limitation of the study

The main limitation of this study was the loss of follow-up. The follow-up of this population is extremely challenging, mainly due to the fact that (1) the majority of the study participants are migrant workers who are highly mobile and (2) they return to their homes, in the most remote areas of the highlands, after the end of the agricultural season.

## STAR★Methods

### Key resources table

**Table:** 

Reagent	Source	Identifier
**Antibodies**		

Anti-human CD4^FITC^	eBioscience	11-0049-80
Anti-human CD8^PE CY7^	eBioscience	25-0088-42
Anti-human CTLA-4^APC^	eBioscience	17-1522-82
Anti-human PD1^PE^	eBioscience	12-9985-82
CD3/CD4/CD8a Antibody Cocktail, APC, FITC, PerCP-eFluor 710	eBioscience	22-0306-71

**Deposited data**		

Custom code	this manuscript	https://github.com/rwomersley/VL_timetorelapse_logreg
RNA-seq sequence data	this manuscript	https://ega-archive.org/

### Resource availability

#### Lead contact

Further information and requests for resources and reagents should be directed to and will be fulfilled by the lead contact, Pascale Kropf (p.kropf@imperial.ac.uk).

#### Materials availability

This study did not generate new unique reagents.

### Experimental model and subject details

A cross-sectional study was conducted with twenty-five healthy male non-endemic controls (median age 28.0 ± 1.3 years old) recruited among the staff of the University of Gondar, Ethiopia; and 99 male patients with visceral leishmaniasis (VL patients) recruited from the Leishmaniasis Treatment and Research Centre (LRTC), University of Gondar. Forty-nine were HIV infected (VL/HIV) (median age 33.5 ± 1.0 years old) and 50 were HIV uninfected (VL) (median age 25 ± 0.8 years old). Patients age < 18 years were excluded. VL and VL/HIV patients were recruited at four different time points: time of diagnosis (ToD); end of treatment (EoT) ; 3 months post the end of treatment (3 m); and 6-12 months post the end of treatment (6-12 m). At the end of this 3-year study, all VL/HIV patients who had not relapsed during the 12 months follow-up were contacted by phone to find out if they had had any further relapse.

This study was approved by the Institutional Review Board of the University of Gondar (IRB, reference O/V/P/RCS/05/1572/2017), the National Research Ethics Review Committee (NRERC, reference 310/130/2018) and Imperial College Research Ethics Committee (ICREC 17SM480). Informed written consent was obtained from each patient and control.

### Method details

#### Diagnosis and treatment of VL and HIV

• - The diagnosis of VL was based on two laboratory tests: 1. Positive serology by using an immuno-chromatographic test, using the recombinant antigen K39, to detect the presence of antibodies against *Leishmania* parasites (IT Leish rapid test, Bio-Rad Laboratories, USA); 2. Positive parasitology: Spleen or bone marrow aspirates from the patients were smeared on a microscope slide, dried and fixed with absolute methanol for 2 minutes. The fixed slides were stained with Giemsa stain for 10 minutes and dried. Diagnosis was confirmed by identifying the amastigote stage of the parasite by microscopy using a 100X objective. The parasite load was graded by following a parasite grading system:
6+: > 100 parasites per field (under a 10X eyepiece and 100X oil-immersion lens),
5+: 10–100 parasites per field, 4+: 1–10 parasites per field, 3+: 1–10 parasites per 10 fields, 2+: 1–10 parasites per 100 fields, 1+: 1–10 parasites per 1000 fields, 0: 0 parasite per 1000 fields.^66^
• The diagnosis of HIV was done at the LRTC by detecting HIV-1 and HIV-2 antibodies, according to the Ethiopian national HIV screening test guideline, by using a screening algorithm using STAT-PAK® (Chembio diagnostics systems. INC, USA), ABON (Abon Biopharm Co., Ltd. China), and SD. BioLine (Standard Diagnostics. INC, India). According to the Ethiopian national HIV screening algorithm, STAT-PAK negative is reported as HIV negative. If STAT-PAK is positive, the sample will be tested with ABON and if ABON is positive the result will be reported as HIV positive. SD. BioLine was used as a tiebreaker when discrepancies appear between the results of STAT-PAK and ABON. This is, if STAT-PAK is positive, ABON is negative and SD. BioLine is positive, the result will be reported as HIV positive. If STAT-PAK is positive, ABON negative, and SD. BioLine negative the result will be reported as negative.
• VL treatment: All treatments (Table S1) were administered according to the Guideline for Diagnosis and Prevention of Leishmaniasis in Ethiopia.^22^ At EoT, all VL patients were clinically cured, as defined by the following criteria: patients look improved, are afebrile, usually have a smaller spleen size and have an improved hematological profile. A test of cure (TOC) was used for VL/HIV patients to assess if they could be discharged from hospital, as defined by the World Health Organization^22^; a negative TOC is defined as follows: patients look improved, afebrile, and usually have a smaller spleen size, parasitological cure (absence of amastigotes in splenic aspirates) and an improved hematological profile. If the TOC was still positive, treatment was continued until TOC becomes negative.

At EoT, tissue aspirates were performed with 5 VL and 17 VL/HIV patients, the results of the microscopy were negative and all patients were discharged from the hospital.

The definitions for no relapse and relapse are defined by the by the “Guidelines for diagnosis, treatment and prevention of leishmaniasis in Ethiopia”^22^ as follows:

no relapse: “absence of clinical features of the disease 6 months after completion of the recommended dose and duration for VL.”
Relapse: “patient with VL treatment history presenting with clinical visceral Leishmaniasis symptoms such as and is diagnosed with positive parasitology (LD bodies) after successful completion of the treatment.”
• HIV treatment: ART was provided according to the National guideline for comprehensive HIV prevention, care and treatment. 46 VL/HIV patients were on ART at the time of VL diagnosis, the remaining three started ART at the end of the anti-leishmanial treatment, all patients were on ART following the Ethiopian national guidelines for comprehensive HIV prevention, care, and treatment, and the following combination therapies were used Tenofovir disoproxil fumarate (TDF) + Lamivudine (3TC) + Efavirenz (EFV), azidothymidine (AZT) + 3TC + EFV or TDF + 3TC + atazanavir (ATV).

#### Sample collection and processing

A total of 13ml of blood was collected by venipuncture from patients and controls. The samples were divided as follows: 2.5ml were added in PAXgene tubes for RNA-Seq. 8ml were added to heparinised tubes: of these 3ml were used for the WBA and 5ml for flow cytometry; and 2.5ml of blood were added into Ethylenediaminetetraacetic acid (EDTA) tubes for complete blood cell count, CD4^+^ and CD8^+^ T cell counts. The tubes were then centrifuged for 5 minutes at 3500 rpm and plasma was collected (from EDTA tubes) for the determination of HIV viral load and for the cytokine profile (from heparinised tubes).

PBMCs were isolated from 5ml of blood was processed within 10minutes after collection using density gradient centrifugation with Histopaque®-1077 (Sigma). 5ml of Histopaque®-1077 was added into 15ml centrifuge tubes and 5ml of blood was layered at the top and centrifuged at 2200rpm for 30minutes with the brake off. At the end of the centrifugation, the plasma at the top layer was separated and stored at −20°C for cytokine and chemokine measurement. PBMCs were collected, washed with phosphate buffer saline (PBS), and used for flow cytometry.^18^

From the purified PBMCs, the espression levels of CTLA-4 and PD1 on CD4^+^ and CD8^+^ T cells was measured by using the following antibodies: CD4^FITC^, CD8^PE CY7^, CTLA-4^APC^ and PD1^PE^ (eBioscience).^18^ The data on PD1 expression are shown as the integrated Median Fluorescent Intensity (iMFI), which reflects the total functional response^67^ more accurately than the frequency of expression.

CD4^+^ and CD8^+^ T cell counts were measured by using 100μl of whole blood collected in EDTA. Cells were stained with CD4^FITC^ and CD3^PerCP-eFluor® 710^, CD8α^APC^ (eBioscience) for 15min at 4°C; red blood cells were lysed using BD FACS Lysing Solution for 5min at room temperature. Acquisition was performed using BD Accuri C6 flow cytometry and data were analyzed using BD Accuri C6 analysis software.

• Whole blood assay: Soluble *leishmania* antigen (SLA) was prepared as follows.^18^ Splenic or bone marrow aspirates were added to 1 mL of the following culture media: 500ml of M199 medium (Sigma, USA) which was enriched with 25mM HEPES, 0.2μM folic acid, 5ml vitamin mix, 1mM hemin, 1mM adenine, 800μM Biopterin, 5ml of penicillin/streptomycin and 50ml fetal bovine serum (Sigma, USA). The culture medium was sterilized by filtration and stored at −20°C until it is used.

The parasites from spleen aspirates of 2 VL and 2 VL/HIV patients were cultured at room temperature and the cultures were monitored under the microscope. The parasites were then pooled and transferred in a new multiple large volume up to 80ml fresh medium and expanded. Stationary-phase promastigotes were harvested and centrifuged at 4500rpm for 20minutes, the pellet was washed three times with cold PBS (Sigma, USA) and counted. The pellet was adjusted to 2 × 10^9^/ml and resuspended in SLA reagent (50mM of EDTA, 50mM of HCl, 100mM of phenylmethanesulfonyl fluoride (PMSF) (Sigma, USA), and 5mg/ml of leupeptin (Sigma, USA). The suspension was sonicated 4-5 times for 15seconds at 10Hz and centrifuged at 27,000xg for 30 minutes at 4°C. The lipid layer was removed from the surface of the supernatant. The remaining supernatant was ultra-centrifuged at 100,000xg for 4hrs at 4°C with the breakoff. The supernatant was collected, the protein concentration was determined, sterile filtered, and the sterile SLA solution was stored at −20°C and used to stimulate whole blood cells in the whole blood assay.

Three milliliters of blood collected in heparinized tubes were used and 1ml aliquots were distributed in 3 tubes and stimulated with SLA (10 μg/ml), Phytohaemagglutinin (PHA, Sigma) (10 μg/ml) and PBS as a negative control. The tubes were incubated for 24hours at 37°C. The tubes were then centrifuged at 3000rpm for 5minutes and supernatants were collected and stored at −20°C.

IFNγ and IL-10 levels were measured in the supernatant of the WBA using IFN gamma and IL-10 Human ELISA Kit (Invitrogen) according to the manufacturer’s instructions. The optical densities obtained with the unstimulated whole blood cells were subtracted from the optical densities obtained with PHA or SLA.

• Inflammatory markers: Plasma was collected after gradient centrifugation of 5ml of heparinised blood as described above in the PBMCs purification^18^ and IFNγ, IL1β, IL2, IL4, IL6, IL8, IL10, IL12p70, IL13, TNFα and CRP plasma levels were measured by multiplex assay using U-PLEX Proinflam Combo 1, V-PLEX Proinflammatory Panel1 Kit and V-PLEX Human CRP following the manufacturer’s instructions (Meso Scale Diagnostics, USA). Levels of IFNα and IFNβ were measured by ELISA (human IFNα ELISA kit, Invitrogen and VeriKine Human IFNβ ELISA KIT, pbl assay science) in the plasma of VL/HIV patients who relapsed and those who didn’t relapse during the follow-up period.
• HIV viral load: Plasma was isolated by centrifuging 2ml of EDTA whole blood and frozen at −80°C. HIV viral load was measured in the Central Laboratory of the Amhara Public Health Institute, Bahir Dar, by using Abbott RealTime HIV-1 Qualitative (m2000sp), according to the manufacturer’s instructions.
• Cell count: White and red blood cell, and platelet counts were measured using a Sysmex XP-300T^M^ automated hematology analyzer, (USA) following the manufacturer’s instruction.
• Organ function tests: Liver and renal function tests were performed as part of the routine management practice. The following reagents (HUMAN Diagnostics) were used according to the manufacturer’s protocol: serum glutamic-oxaloacetic transaminase (SGOT liquiUV) and serum glutamic pyruvic transaminase (SGPT liquiUV); blood urea nitrogen (BUN) (Urea liquicolor test) and creatinine (creatinine liquicolor test) with a detection limit of SGOT = up to 500U/L, SGPT = up to 500U/L, BUN = up to 200mg/dl and creatinine = up to 13mg/dl. Concentration was measured by AMS VEGASYS Fully Automated chemistry analyzer (Italy).
• mRNA: 2.5 mL of blood was collected in PAXgene blood RNA tubes, RNA extracted using the PAXgene 96 blood RNA kit (QIAGEN) and Globin mRNA depleted using the GLOBINclear kit (Ambion). Sequencing libraries were prepared using the KAPA Stranded mRNA-Seq Kit (Roche) with 10 PCR cycles, then sequenced as 75bp paired-end reads on the Illumina HiSeq 4000 platform. Sequencing reads were mapped with Salmon v.1.30 against concatenated sequence of human gencode transcriptome release 34, transcripts for *L. donovani* LV9 from TriTrypDB release 46 and transcript data for an Ethiopian HIV type 1C virus (GenBank Accession U46016) - type 1C represents over 90% of HIV infections in Ethiopia. Pseudo-counts were imported into R v4.0.3 using the tximport v1.18.0 and transformed into lengthscaledTPM. Total HIV expression was quantified as the sum of counts across HIV transcripts, and total *Leishmania* expression was quantified as the sum across all LV9 transcripts with the exception of feature LdLV9.27.2.206410, an 18S rRNA gene to which human transcripts also map.

### Quantification and statistical analysis

Data were evaluated for statistical differences as specified in the legend of each figure. The following tests were used: Mann-Whitney, Kruskal-Wallis or Spearman’s rank test. Differences were considered statistically significant at p < 0.05. Unless otherwise specified, results are expressed as median ± SEM. ∗ = p < 0.05, ∗∗ = p < 0.01, ∗∗∗ = p < 0.001 and ∗∗∗∗ = p < 0.0001.

• Relapse rate: Log-rank tests for duration of follow-up at event end points provided two-sided *p*-values; Kaplan-Meier curves are presented for visual interpretation. The primary outcome survival until three years of follow-up was completed; relapse was the only censoring event. Censoring events were reported at the pre-planned follow-up period (3, 6-12 and after 3 years) at which they were identified. Cox proportional-hazards regression analysis was used to estimate hazard ratios and 95% confidence intervals.
• Logistic regression: A multinomial logistic regression analysis was performed in R (version 4.0.3) (R Core Team, 2020), using raw CD4 counts, IFNγ and PD1 measured at EoT to predict relapse time for individuals. After removing missing data, a cohort of 34 individuals were allocated to three potential categories based on time (in months) to relapse after EoT: “Relapse at 3m” (n = 10), “Relapse at 6-12m,” (n = 7) and “No Relapse in Study” (n = 17). Performance of the model of best fit was assessed by plotting a receiver operating curve (ROC) for predicting relapse at 3 m and 6-12 m. Area under the ROC curve (AUC) was then calculated with a 95% confidence interval (CI) for both groups.

## Data Availability

All data reported in this paper will be shared by the lead contact upon request. RNA-seq sequence data is available from the European Genome-Phenome Archive (https://ega-archive.org/). Accession numbers are shown in Table S1. The script that contains the data and model building and evaluation of a multinomial logistic regression model have been deposited at GitHub; https://github.com/rwomersley/VL_timetorelapse_logreg. Any additional information required to reanalyse the data reported in this paper is available from the lead contact upon request.

## Supplemental information

Document S1. Figures S1–S5 and Tables S1–S4

Document S2. Article plus supplemental information
