# Supplementary material for: Immunological factors, but not clinical features, predict visceral leishmaniasis relapse in patients co-infected with HIV
Source: Cell Rep Med. 2021 Dec 30;3(1):100487. doi: 10.1016/j.xcrm.2021.100487 (PMC8784791; doi:10.1016/j.xcrm.2021.100487)
Supplement: Document S1. Figures S1–S5 and Tables S1–S4 [file mmc1.pdf]

Cell Reports Medicine, Volume *3*

# Supplemental information

# Immunological factors, but not clinical features, predict visceral leishmaniasis relapse in patients co-infected with HIV

**Yegnasew Takele, Tadele Mulaw, Emebet Adem, Caroline Jayne Shaw, Susanne Ursula Franssen, Rebecca Womersley, Myrsini Kaforou, Graham Philip Taylor, Michael Levin, Ingrid Müller, James Anthony Cotton, and Pascale Kropf**

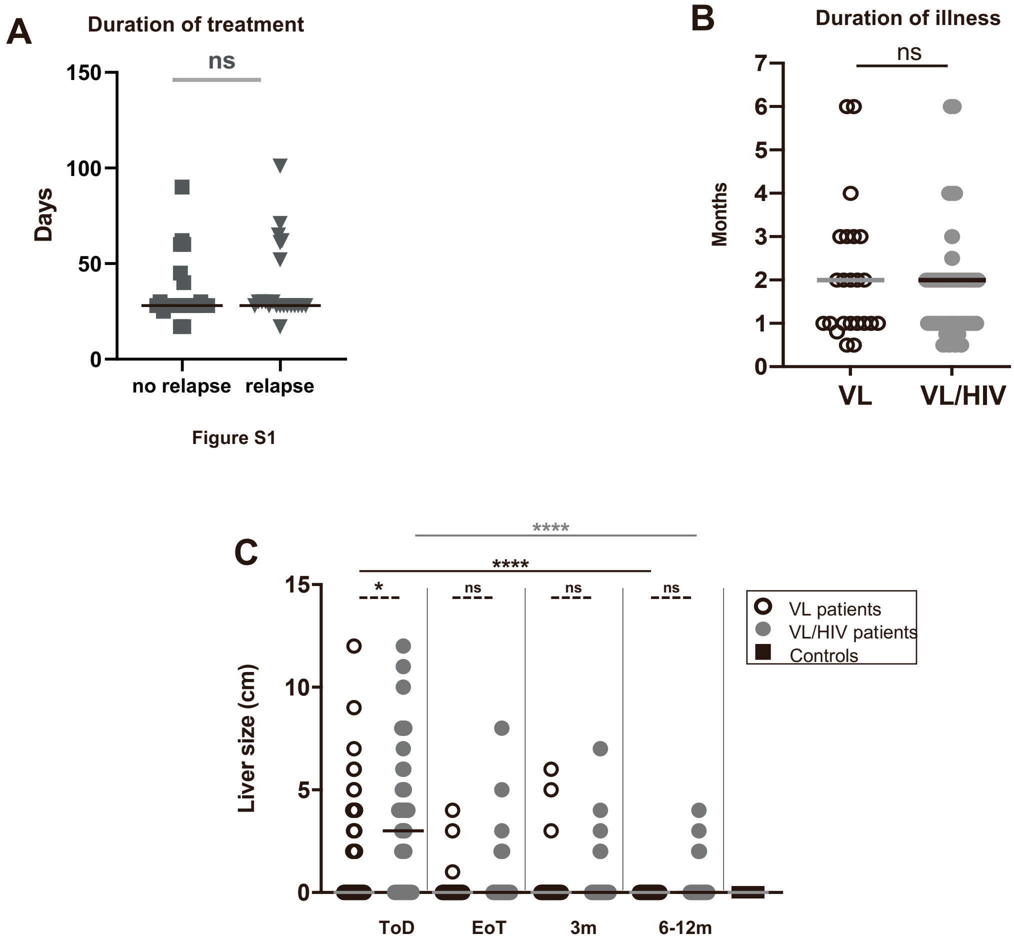


**Figure S1: Duration of treatment, illness and liver size, related to Table 1: A.** comparison of the duration of initial VL treatment (at ToD) between VL/HIV patients who relapsed (n=20) and those who didn't relapse during follow-up (n=17). **B.** comparison of the duration of illness as defined by the duration of time since the patients have started experiencing symptoms such as enlarged abdomen, fever, fatigue and loss of weight. **C.** liver size was measured in cm below the costal margin on VL (ToD: n=50, EoT: n=45, 3m: n=36, 6-12m: n=26), VL/HIV (ToD: n=49, EoT: n=39, 3m: n=32, 6-12m: n=26) patients and controls (n=25) and controls (n=25).

Each symbol represents the value for one individual, the straight lines represent the median. Statistical differences between VL and VL/HIV patients at each time point or between no relapse and relapse were determined using a Mann-Whitney test and statistical differences between the 4 different time points for each cohort of patients were determined by Kruskal-Wallis test.

ToD=Time of Diagnosis; EoT=End of Treatment; 3m=3 months post EoT; 6-12m=6-12 months post EoT. ns=not significant.

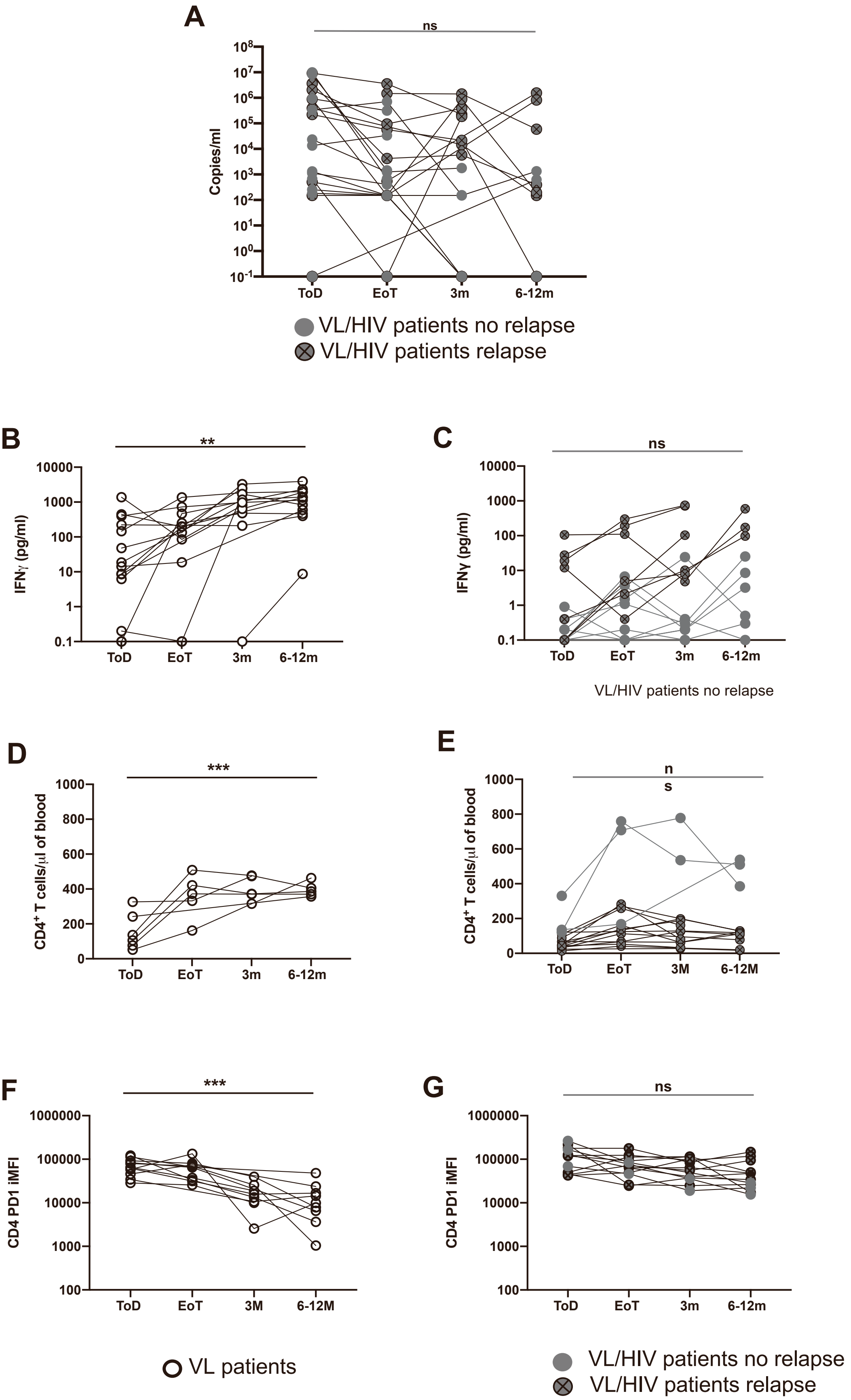


**Figure S2: Longitudinal analyses of viral loads, IFNγ , CD4$^{+}$ T cell counts and CD4 PD1 iMFI: A.** viral load (copies/ml) were measured longitudinally in the plasma of 35 VL/HIV patients who had at least 2 measurements of their viral load over the duration of the study, related to Table 1. **B**. Whole blood cells from VL patients (ToD: n=14, EoT: n=11, 3m: n=11, 6-12m: n=12) and **C.** VL/HIV patients (ToD: n=17, EoT: n=16, 3m: n=16, 6-12m: n=9) were cultured in the presence of SLA and IFNγ was measured by ELISA in the supernatant after 24hrs, related to Figure 2A. **D.** CD4$^{+}$ T cell counts were measured by flow cytometry in the blood of VL (ToD: n=6, EoT: n=5, 3m: n=5, 6-12m: n=5) and **E.** VL/HIV patients (ToD: n=16, EoT: n=12, 3m: n=13, 6-12m: n=12), related to Figure 3A. **F.** CD4 PD1 iMFI was measured by flow cytometry in the PBMCs of

VL (ToD: n=11, EoT: n=10, 3m: n=8, 6-12m: n=9) and **G.** VL/HIV patients (ToD: n=11, EoT: n=12, 3m: n=11, 6-12m: n=11), related to Figure 3E.

Grey full circle=VL/HIV who did not relapse during the study. Grey full circle with a cross=VL/HIV who relapsed during the study. Data are shown for patients who had at least 2 measurements during the course of the study. Statistical differences were determined using a one-way ANOVA. ns=not significant. ToD=Time of Diagnosis; EoT=End of Treatment; 3m=3 months post EoT; 6-12m=3 months post EoT. ns=not significant.

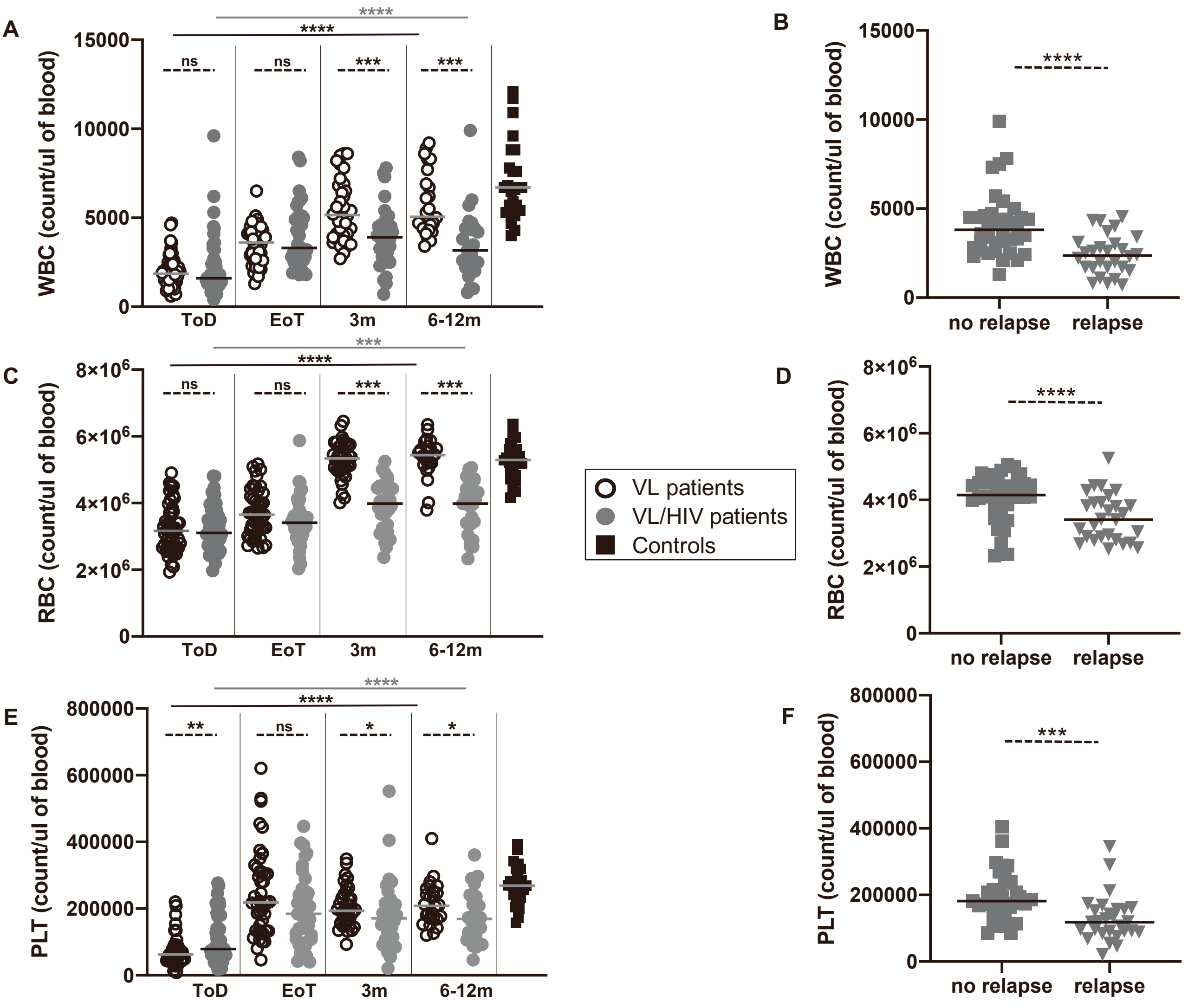


**Figure S3: White and red blood cells and platelet counts, related to Figure 1: A.** White blood cells, **C.** Red blood cells and **E.** Platelet counts in the blood of VL (ToD: n=50, EoT: n=45, 3m: n=36, 6-12m: n=26), VL/HIV (ToD: n=49, EoT: n=39, 3m: n=32, 6-12m: n=27) patients and controls (n=25). **B.** White blood cells, **D.** Red blood cells and **F.** Platelet counts in the blood of VL/HIV patients who did not relapse (n=34) and those who relapsed (n=28) after successful anti-leishmanial treatment, during the 3m and 6-12 follow-up period. If a patient did not relapse during the two-time points of follow-up and if a patient relapsed at both 3 and 6-12 months, this is represented as 2 measurements.

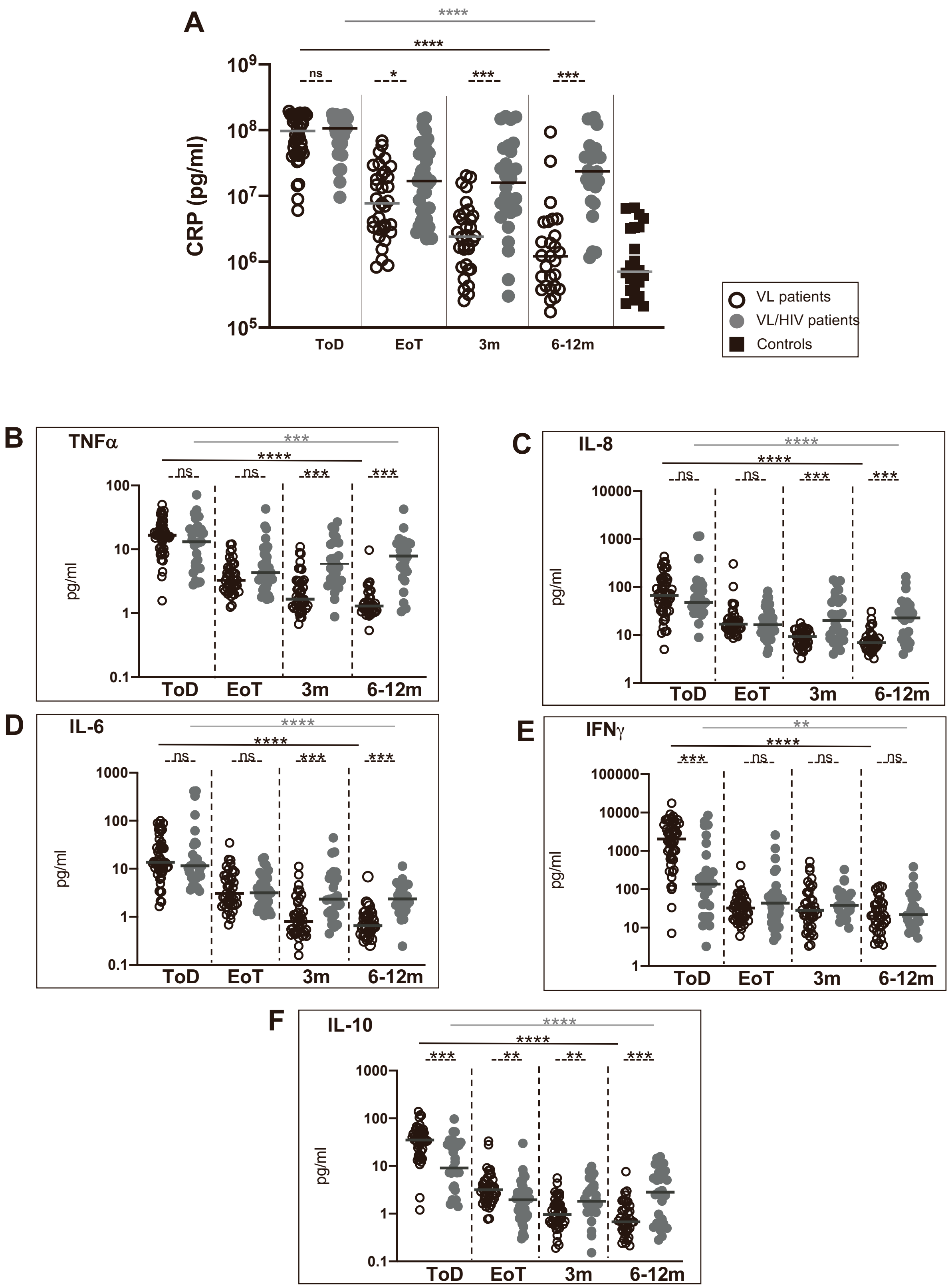


**Figure S4: CRP, pro- and anti-inflammatory cytokines, related to Figure 2: A.** CRP levels were measured in the plasma isolated from the blood of VL (ToD: n=42, EoT: n=33, 3m: n=32, 6-12m: n=27), VL/HIV patients (ToD: n=29, EoT: n=39, 3m: n=28, 6-12m: n=28) and controls (n=22) by multiplex assay. Levels of **B.** TNFα; **C.** IL-8, **D.** IL-6; **E.** IFNγ and **F.** IL-10 were measured in the plasma isolated from the blood of VL (ToD: n=42, EoT: n=33, 3m: n=32, 6-12m: n=27), VL/HIV patients (ToD: n=29, EoT: n=39, 3m: n=28, 6-12m: n=28) and controls (n=22) by multiplex assay.

Each symbol represents the value for one individual, the straight lines represent the median. Statistical differences between VL and VL/HIV patients at each time point or between no relapse and relapse were determined using a Mann-Whitney test and statistical differences between the 4 different time points for each cohort of patients were determined by Kruskal-Wallis test. ToD=Time of Diagnosis; EoT=End of Treatment; 3m=3 months post EoT; 6-12m=3 months post EoT. ns=not significant.

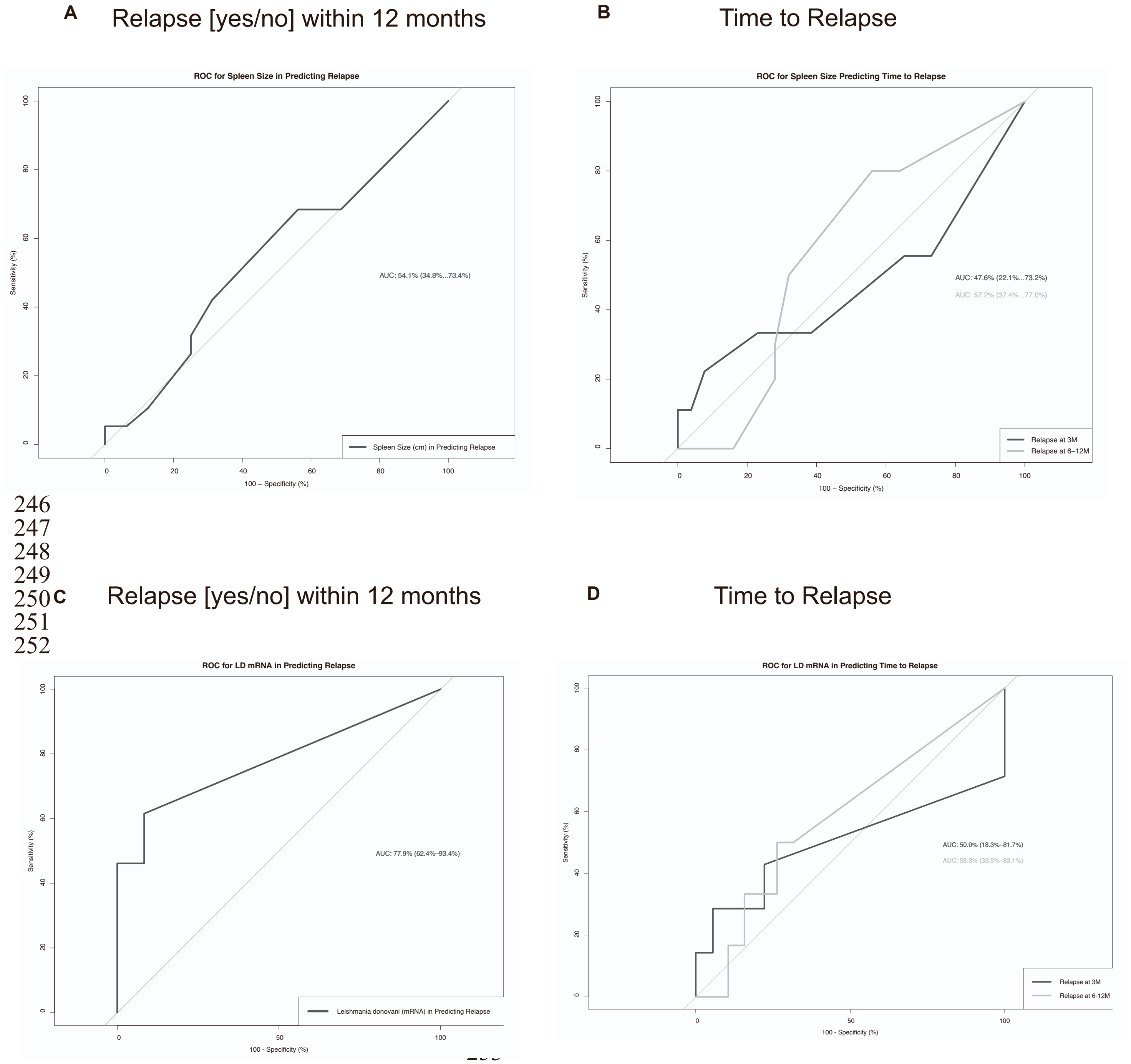


**Figure S5, related to Figure 4: ROC curve: spleen size and *Ld* mRNA.** Spleen size ROC curve for predicting VL relapse **A.** at 3m and **B.** 6-12m groups for VL/HIV individuals. *Ld* mRNA ROC curve for predicting VL relapse **C.** at 3m and relapse **D.** at 6-12m groups for VL/HIV individuals.

**Table S1: Treatments, related to Figure 1**

| Number of VL patients | Antileishmanial treatment | Duration of treatment in days |
|---|---|---|
| 39 | SSG+PM | 17 |
| 1 | SSG+PM | 30 |
| 7 | AmBisome | 28 |
| 2 | SSG | 28 |
| 1 | SSG+PM | Treatment not completed |
| | | |
| **Number of VL/HIV patients** | **Antileishmanial treatment** | **Duration of treatment in days** |
| 2 | SSG+PM | 61 |
| 4 | SSG | 28 |
| 1 | SSG | 71 |
| 1 | AmBisome | 60 |
| 6 | AmBisome | 28 |
| 2 | AmBisome | 65 |
| 17 | AmBisome + Miltefosine | 28 |
| 1 | AmBisome + Miltefosine | 40 |
| 2 | AmBisome + Miltefosine | 45 |
| 2 | AmBisome + Miltefosine | 62 |
| 1 | AmBisome + Miltefosine | 90 |
| 1 | AmBisome + Miltefosine | 52 |
| 1 | AmBisome + Miltefosine | 101 |
| 2 | AmBisome + pentamidine | 30 |
| 2 | AmBisome + pentamidine | 62 |
| 4 | AmBisome | Treatment not completed |

SSG= Sodium stibogluconate, PM= Paramomicin and AmBisome= Liposomal amphotericin B.

**Table S2: Treatment type for VL/HIV patients who required a longer treatment, related to Figure 1**

| Number of patients with no relapse | Type of treatment | Number of patients with relapse | Type of treatment |
|---|---|---|---|
| 2 | AmBisome | - | AmBisome |
| 2 | AmBisome + Miltefosine | 4 | AmBisome + Miltefosine |
| 1 | SSG+PM | 1 | SSG+PM |
| - | AmBisome + pentamidine | 1 | AmBisome + pentamidine |
| 1 | SSG | - | SSG |

List of the different initial treatments used to treat VL in VL/HIV patients who went on to relapse and those who did not relapse. SSG= Sodium stibogluconate, PM= Paramomicin and AmBisome= Liposomal amphotericin B.

**Table S3: Comparison of clinical, haematological and immunological parameters at EoT between VL/HIV patients who went onto relapse and those who did not relapse during follow-up, related to Figure 1A**

| **Parameters** | **No relapse** | **Relapse** | ***p* value** |
|---|---|---|---|
| LD mRNA | 0.1±0.1 | 1.0 ±341.4 | *p*=0.0022 |
| Viral load (copies/µl) | 278±39224 | 150±196589 | *p*=0.7519 |
| Body temperature (ºC) | 36.3±0.1 | 36.4±0.1 | *p*=0.9705 |
| Spleen size (cm) | 2.5±0.7 | 3.0±0.7 | *p*=0.5064 |
| Liver size (cm) | 0.0±0.4 | 0.0±0.2 | *p*=0.8895 |
| BMI | 16.8±0.4 | 17.3±0.4 | *p*=0.4926 |
| WBCs (count/µl) | 3250 ± 415 | 3300 ± 378 | *p*=0.8933 |
| RBCs (count/µl) | 3380000 ± 153499 | 3410000 ± 166189 | *p*=0.6669 |
| Platelets (count/µl) | 203000 ± 30919 | 172000 ± 20055 | *p*=0.7670 |
| WBA IFNγ (PHA) (pg/ml) | 31.2±130.7 | 18.6±181.1 | *p*=0.8397 |
| WBA IL-10 (SLA) (pg/ml) | 0.3±4.2 | 1.9±1.5 | *p*=0.4261 |
| WBA IL-10 (PHA) (pg/ml) | 186.0±62.0 | 231.1± 61 2 | *p*=0.7521 |
| $CD8^+$ T cell count | 399±75 | 287.7±54.0 | *p*=0.1559 |
| TNF-α (pg/ml) | 4.8±2.8 | 4.1±0.6 | *p*=0.3147 |
| IL-8 (pg/ml) | 19.2±2.8 | 15.9±4.3 | *p*=0.4041 |
| IL-6 (pg/ml) | 2.6±1.2 | 3.6±0.7 | *p*=0.4756 |
| IFNγ (pg/ml) | 41.6±69.9 | 43.5±12.9 | *p*=0.4041 |
| IL-10 (pg/ml) | 2.2±1.8 | 1.9±1.2 | *p*=0.4041 |
| CRP (pg/ml) | 10013339 ± 12128126 | 34335,105 ± 9737453 | *p*=0.1011 |

Clinical, haematological and immunological parameters were measured at EoT for VL/HIV patients who went onto relapse and those who did not relapse during follow-up. Statistical differences between no relapse and relapse were determined using a Mann-Whitney test.

**Table S4: IFNα and IFNβ in the plasma of VL/HIV patients with and without relapse, related to Figure 1A**

| | **no relapse** | **relapse** | ***p* value** |
|---|---|---|---|
| **IFNα** | 0.0±1.9 | 0.0±8.1 | 0.2156 |
| **IFNβ** | 0.0±0.7 | 0.0±7.7 | 0.4177 |

Levels of IFNα and IFNβ were measured by ELISA in the plasma isolated from the blood of VL/HIV patients who did not relapse and those who went on to relapse during the 3m and 6-12 follow-up period. Statistical differences between no relapse and relapse were determined using a Mann-Whitney test.
